# Regulation of RAB5C Is Important for the Growth Inhibitory Effects of MiR-509 in Human Precursor-B Acute Lymphoblastic Leukemia

**DOI:** 10.1371/journal.pone.0111777

**Published:** 2014-11-04

**Authors:** Yee Sun Tan, MinJung Kim, Tami J. Kingsbury, Curt I. Civin, Wen-Chih Cheng

**Affiliations:** 1 Center for Stem Cell Biology & Regenerative Medicine, University of Maryland School of Medicine, Baltimore, Maryland, United States of America; 2 Greenebaum Cancer Center, University of Maryland School of Medicine, Baltimore, Maryland, United States of America; 3 Department of Physiology, University of Maryland School of Medicine, Baltimore, Maryland, United States of America; 4 Department of Pediatrics, University of Maryland School of Medicine, Baltimore, Maryland, United States of America; University of Sydney, Australia

## Abstract

MicroRNAs (miRs) regulate essentially all cellular processes, but few miRs are known to inhibit growth of precursor-B acute lymphoblastic leukemias (B-ALLs). We identified miR-509 via a human genome-wide gain-of-function screen for miRs that inhibit growth of the NALM6 human B-ALL cell line. MiR-509-mediated inhibition of NALM6 growth was confirmed by 3 independent assays. Enforced miR-509 expression inhibited 2 of 2 additional B-ALL cell lines tested, but not 3 non-B-ALL leukemia cell lines. MiR-509-transduced NALM6 cells had reduced numbers of actively proliferating cells and increased numbers of cells undergoing apoptosis. Using miR target prediction algorithms and a filtering strategy, RAB5C was predicted as a potentially relevant target of miR-509. Enforced miR-509 expression in NALM6 cells reduced RAB5C mRNA and protein levels, and RAB5C was demonstrated to be a direct target of miR-509. Knockdown of RAB5C in NALM6 cells recapitulated the growth inhibitory effects of miR-509. Co-expression of the RAB5C open reading frame without its 3′ untranslated region (3′UTR) blocked the growth-inhibitory effect mediated by miR-509. These findings establish RAB5C as a target of miR-509 and an important regulator of B-ALL cell growth with potential as a therapeutic target.

## Introduction

More effective and less toxic therapies are needed for precursor-B acute lymphoblastic leukemia (B-ALL), the most common childhood cancer [1]–[3]. To find novel therapeutic targets, deeper understanding of the mechanisms involved in leukemia cell proliferation and survival is necessary. MicroRNAs (miRs) are short non-coding RNAs which regulate expression of mRNA targets, most commonly by binding to the 3′ untranslated regions (3′UTRs) of mRNAs [4]–[6]. Each miR has many, often hundreds of predicted mRNA targets, and reciprocally a single mRNA may be targeted by multiple miRs. MiRs are involved in many cellular processes, and dysregulation of miRs has been linked to diseases, prominently including cancer [7]. For instance, overexpression of miR-155 has been detected in certain subtypes of acute myeloid leukemia (AML), chronic lymphoblastic leukemia, and lymphomas [8]. Transplantation of mouse bone marrow cells overexpressing miR-155 resulted in myeloproliferative disorders, and transgenic overexpression of miR-155 resulted in ALL and lymphoma in mice [9], [10]. In contrast, miR-34 is a well-studied tumor suppressor miR; its expression is down-regulated in a wide range of solid and hematologic malignancies, and it targets multiple molecules that promote cancer development and progression, including BCL2 and cyclin D1 [11], [12].

Expression profiling studies, such as microarray hybridization, real-time PCR, or sequencing assays of global miR expression in leukemia cells versus normal counterpart cells, are often used to identify miRs associated with acute leukemias [13]–[15]. In B-ALLs, multiple miRs are known to be dysregulated [16], [17], but only a few miRs, including miR-196b [18], miR-124a [19] and miR-143 [20], have been shown to inhibit B-ALL growth. Although expression profiling studies can implicate miRs as biomarkers, it is often difficult to differentiate ‘passenger miRs’ from ‘driver miRs’ [21]. As an alternative to expression profiling approaches, functional screens for miRs that drive hallmark cancer properties have successfully identified miRs involved in regulation of cellular processes including growth in melanoma [22], pancreatic cancer [23], and colon cancer [24], as well as metastasis in liver cancer [25]. We previously identified a set of miRs that regulate growth of the human lung fibroblast cell line IMR90 by a miR-high throughput functional screen (miR-HTS) [26]. In this paper, we extended our gain-of-function screening of human miRs to B-ALL cells and identified miR-509 as a novel B-ALL growth-inhibitory miR. MiR-509 inhibited growth of 2 additional B-ALL cell lines. We went on to determine the cellular mechanism of miR-509-mediated B-ALL growth inhibition and identify RAB5C as a key B-ALL growth-promoting factor targeted by miR-509.

## Material and Methods

### Functional screen of miRs

Detailed description of the miR-HTS methodology was previously described [26]. Briefly, in each miR-HTS, 1.8 million NALM6 cells were infected at a multiplicity of infection (MOI)  = 0.3 with the human Lenti-miR pooled virus library (System Biosciences, Mountain View, CA, USA; Cat# PMIRHPLVA-1) to achieve ∼30% transduced cells. 4 µg/ml polybrene (Sigma-Aldrich, St. Louis, MO, USA) was used as the infection vehicle. On days 4, 12, 20 and 28 after infection, a fraction of the infected culture (2 million cells) was harvested and genomic DNA isolated using the DNeasy Blood & Tissue Kit (Qiagen, Valencia, CA, USA). To identify candidate growth-regulatory miRs, nested PCR, customized qPCR assays, and candidate selection were conducted as described [26]. 3 independent miR-HTS was conducted.

### Cell lines

NALM6, RCH-ACV, REH, KARPAS-45 were obtained from DSMZ (Braunschweig, Germany). Jurkat and K562 cells were obtained from ATCC (Manassas, VA, USA). All cell lines were maintained according to manufacturer's protocol.

### Plasmids and cloning

Overexpression of miRs was achieved by cloning each precursor miR sequence plus ∼200 bp of flanking genomic sequence into the pJET1.2 plasmid (Thermo Scientific, Waltham, MA, USA) (Primers listed in Table S1). The genomic sequence of each miR was obtained from the UCSC genome browser. The miR sequences were then subcloned into our pWCC52 lentiviral vector (Empty lentiviral vector #1, EV#1) downstream of GFP driven by human EF1α promoter. MiR-509 was also subcloned into our pWCC72 lentiviral vector (empty lentiviral vector #2, EV#2) downstream of DsRed driven by human EF1α promoter. Both pWCC52 and pWCC72 were generated in our lab based on lentivectors designed to express miRs as described [27].

3 plasmids, each containing a different shRNA targeting RAB5C [shRNA#1 (TRCN0000072935), shRNA#2 (TRCN0000072933), shRNA#3 (TRCN0000072937)], were purchased from Thermo Scientific. The plasmid containing non-targeting scramble control sequence was purchased from Addgene (plasmid 1864) [28]. Next, each of the shRNA plasmids was digested with *Bam*HI and *Nde*I to subclone the scramble control sequence and the shRNA containing sequences into pLKO.3G lentiviral plasmid (Addgene Plasmid 14748).

For luciferase assays, full length RAB5C 3′UTR was PCR amplified using cDNA of NALM6 as template, and cloned into pmirGLO Dual-Luciferase miRNA Target Expression vector (Promega, Madison, WI, USA). Site directed mutagenesis of RAB5C-3′UTR-luciferase deletion construct 1 (Δ1) was carried out using the QuikChange Lightning Site-Directed Mutagenesis Kit (Agilent Technologies, Santa Clara, CA, USA) according to manufacturer's protocol. For deletion of the second miR-509-3p binding site in Δ2 construct and Δ1Δ2 constructs, standard PCR was performed. Primers used to create the luciferase constructs are listed in Table S2.

A lentivector overexpressing the RAB5C was constructed by PCR amplification of the RAB5C open reading frame from NALM6 cDNA (Primers listed in Table S3). The PCR product was then cloned into the pWCC61 plasmid (Empty lentiviral vector #3; EV#3), a dual-promoter lentiviral vector generated by our lab in which the human EF1α promoter drives RAB5C and the ubiquitin promoter drives DsRed.

### Lentivirus production and transduction

Lentivector plasmids were co-transfected with purchased packaging plasmids, pMD2.G (Addgene plasmid 12259) and pCMVR8.74 (Addgene plasmid 22036), using 3 µg of polyethylenimine (Polysciences Inc., Warrington, PA, USA) per µg of DNA. Viruses were then titered in each cell line 3 days after transduction by measuring %GFP^+^ cells using flow cytometry. Cultures transduced between 30-70% GFP^+^ were used to calculate lentivirus titer and MOI. To increase transduction efficiency, the following amounts of polybrene was added to each cell line: 0.8 µg/ml polybrene for RCH-ACV and KARPAS-45 cells, 1.6 µg/ml polybrene for Jurkat cells, 4 µg/ml polybrene for NALM6, REH and K562 cells. Mock-transduced cells were cells treated with polybrene but no lentivirus. In all experiments with transduced cells, cells were transduced with each lentivirus to MOI  = 2. All transduced cells were washed with phosphate buffered saline (PBS) at 2 days after transduction to remove the polybrene.

### GFP competition assay

3 days after transduction, >80% of NALM6 cells were GFP^+^. 7 days after transduction, the transduced cells were mixed with mock-transduced cells to obtain a cell mixture containing ∼50% GFP^+^ cells, and this time point was set as day 0 for the GFP competition assay. This cell mixture was cultured for 5 weeks, and the %GFP^+^ cells was measured weekly by flow cytometry (Accuri C6, Becton Dickinson, New Jersey, USA), after gating on only the viable cell population based on the FSC and SSC parameters. Analysis was performed using FlowJo software (Tree Star Inc, Ashland, OR, USA).

### Cell growth assays

For alamarBlue (Life Technologies, Grand Island, NY, USA) dye-based cell growth assays, cells were seeded at 5×10^3^ cells/100 µl media (NALM6 and RCH-ACV cells) or at 2×10^3^ cells/100 µl media (REH cells) in triplicates in 96-well plates at 3 days after transduction. At 7 days after transduction, 10 µl alamarBlue was added to each well and plates incubated (37°C, 4 h) before reading using a VictorX3 (PerkinElmer, Waltham, MA, USA; 530/580 nm excitation/emission filters). For trypan blue exclusion cell counts, 2.5×10^5^ cells/ml were seeded in each well of a 96-well plate day on day 3 after transduction. 10 µl of cell suspensions were removed at each time point and counted using a hemocytometer.

### RNA isolation and measurement of miR and mRNA expression levels by quantitative real-time reverse-transcription PCR (qRT-PCR)

For qRT-PCR of mature miRs, cell lysates were made using Cell Lysis Buffer (Signosis, Santa Clara, CA, USA) and reverse transcription performed using TaqMan microRNA reverse transcription kit (Life Technologies) according to manufacturer's protocol. For mRNA levels, SYBRGreen qRT-PCR assays were conducted with total RNA isolated using the miRNeasy kit (Qiagen) according to manufacturer's protocol, and reverse transcription performed using the High-capacity-RNA-to-cDNA kit (Life Technologies) according to manufacturer's protocol. Primers for qRT-PCR for genes were obtained from PrimerBank [29] (Table S4). The TaqMan IDs are listed in Table S5 (Life Technologies). All SYBRGreen and TaqMan qRT-PCR assays were performed using the 7900 HT Real-Time PCR system (Life Technologies). All Ct values >35 were assigned a value of 35 for calculation of fold expression level change. For qRT-PCR of mature miRs, U18 was used as endogenous control. For SYBRGreen qRT-PCR of mRNA genes, GAPDH was used as endogenous control. DNA oligonucleotides (synthesized by Integrated DNA Technologies, Coralville, IA, USA) of mature miR sequences (miRBase.org) were used to create standard curves for absolute qRT-PCR miR quantitation, which was performed as described previously [30], [31].

### Microarray data

All microarray data has been previously deposited in NCBI Gene Expression Omnibus [32] (GEO Series accession number GSE51908; http://www.ncbi.nlm.nih.gov/geo/query/acc.cgi?acc=GSE51908). Samples used in this analysis include B-ALL cell lines (*n* = 27, replicates of 9 cell lines), primary B-ALL samples (*n* = 16), T-ALL cell lines (*n* = 15, replicates of 5 cell lines), primary T-ALL samples (*n* = 8), AML cell lines (*n* = 21, replicates of 7 cell lines), primary AML samples (*n* = 15), primary blood B lymphocytes (*n* = 11), primary mobilized blood CD34^+^ hematopoietic stem-progenitor cells (HSPCs) (*n* = 4), primary blood granulocytes (*n* = 14), primary blood monocytes (*n* = 5) and primary blood T lymphocytes (*n* = 20).

### Apoptosis and cell cycle analysis

For apoptosis assays, 10^5^ NALM6 cells were stained with APC Annexin V and DNA binding dye 7-amino-actinomycin (7-AAD) (Biolegend, San Diego, CA, USA) 4 days after transduction according to manufacturer's protocol and analyzed by flow cytometry (Accuri C6, Becton Dickinson). For cell cycle analysis, at 3 days after transduction, NALM6 cells (0.5×10^6^ cells/ml) were cultured for 24 h in fresh medium, then 10^6^ cells were labeled with BrdU (Becton Dickinson) for 1 h. Cells were then washed twice in ice cold PBS and the pellet suspended in 500 µl PBS. Cells were fixed in 5 ml ice cold 70% ethanol overnight at −20°C. 2 M hydrochloric acid was then used to denature the DNA for 30 min at room temperature, and the washed pellet resuspended in 1 ml 0.1 M Na_2_B_4_O_7_, pH 8.5 (Sigma-Aldrich) to neutralize the acid for 10 min. Cells were stained with 1 µl APC anti-BrdU antibody (BioLegend) in 20 µl volume for 30 min at room temperature, followed by 20 µl 7-AAD for 15 min at room temperature. APC BrdU and 7-AAD signal was then assessed by flow cytometry (Accuri C6, Becton Dickinson). FlowJo software (Tree Star Inc) was used to determine the cell cycle profile of each sample.

### Caspase-3/7 assay

Transduced NALM6 cells were seeded at 500 cells/well in a 384-well plate on day 3 after transduction. On day 7 after transduction, caspase activity was measured using the Apo-ONE homogenous caspase-3/7 assay (Promega) according to manufacturer's instructions at 4 h after addition of reagent to cells, using a VictorX3 (PerkinElmer, 485/535 nm excitation/emission filters).

### Luciferase assay

HEK293T cells were cultured overnight at 10^5^ cells/450 µl in each well of a 24-well plate. 300 ng of plasmid was co-transfected with 50 nM of miR mimic using 2.5 µl of Lipofectamine2000 (Life Technologies) according to manufacturer's protocol. Lysates were harvested 48 h after transfection and processed using Dual luciferase reporter assay system (Promega) according to manufacturer's protocol. Lysates were diluted 400-fold in passive Lysis buffer Assay before plating and read using VictorX3 (PerkinElmer). Renilla luciferase values were used to normalize for transfection efficiency; the ratio of firefly/renilla luciferase is designated as relative luciferase activity.

### Western blotting

Lysates of transduced cells were harvested 7 days after transduction and lysed in RIPA buffer (Sigma-Aldrich) containing 1 mM phenylmethanesulfonyl fluoride (Sigma-Aldrich) and 1× complete protease inhibitor cocktail tablet (Roche Applied Science, Indianapolis, USA). Protein concentration was determined by Bio-Rad Protein assay (Bio-Rad, Hercules, CA, USA) according to manufacturer's protocol and lysates containing 30-40 µg protein loaded onto a pre-made 4-12% Bis-Tris NuPAGE gel (Life Technologies) and transferred to a PVDF membrane using an iBlot Dry Blotting system (Life Technologies). RAB5C (ab137919, Abcam, Cambridge, MA, USA) and α-tubulin (T6074, Sigma-Aldrich) antibodies were used according to manufacturer's protocol and signal detected using an ECL detection kit (Thermo Scientific) imaged by the ChemiDOC XRS+ System (Bio-Rad). Bands were analyzed and quantified using ImageLab software (Bio-Rad).

## Results

### Enforced miR-509 expression inhibited growth of NALM6 cells

**Figure 1:**
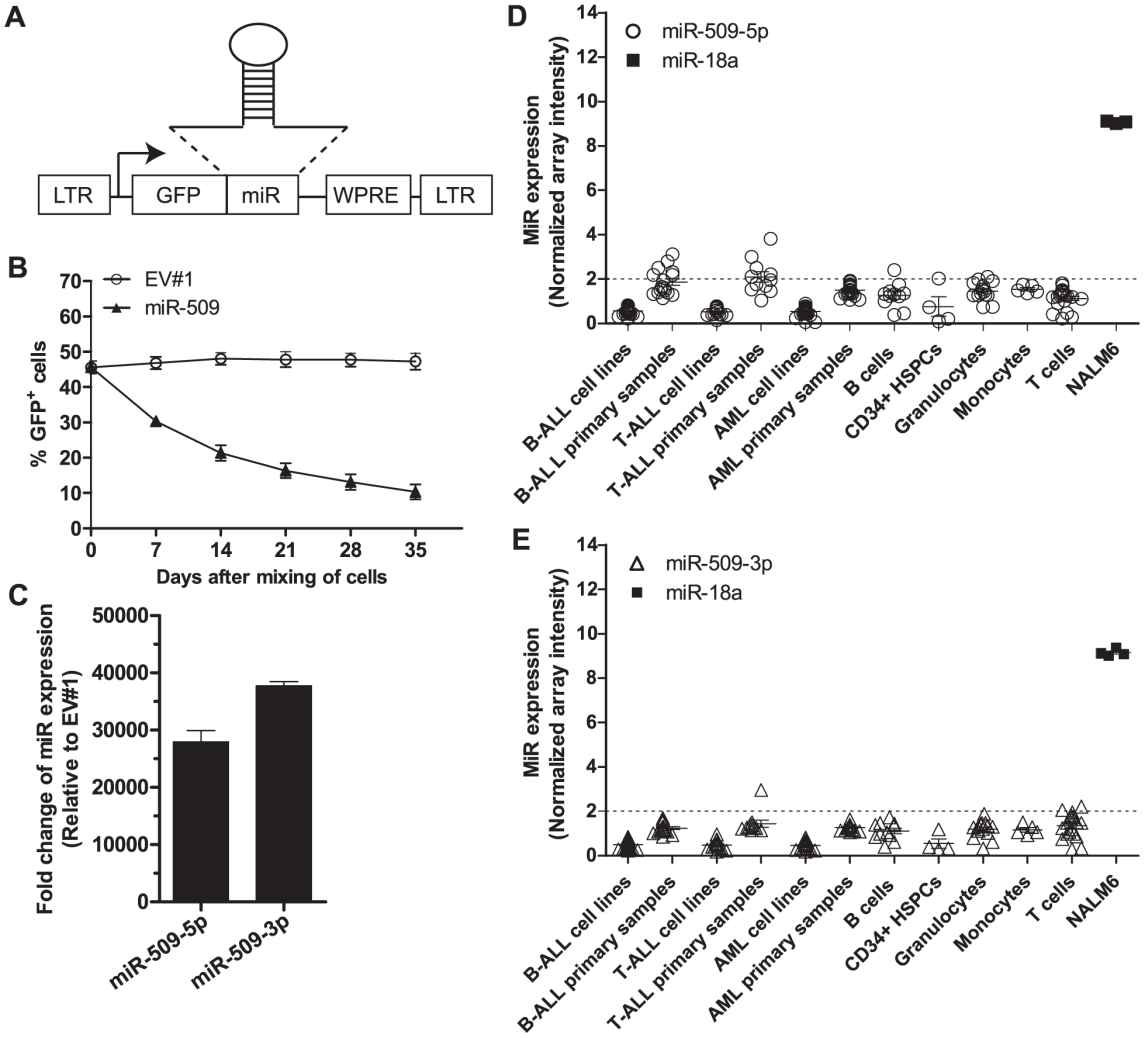
Enforced miR-509 expression inhibits growth of NALM6 cells. (A) Schematic of lentiviral vector used to express miRs. Arrow depicts the direction of human EF1α promoter. LTR: long terminal repeat; GFP: green fluorescent protein; WPRE: woodchuck hepatitis virus post-transcriptional regulatory element. The parental plasmid without miR is denoted as empty vector #1 (EV#1). The miR sequence consists of the native miR hairpin with ∼200 bp of its flanking genomic sequences. (B) Assessment of %GFP^+^ cells by flow cytometry in the GFP competition assay. NALM6 cells were transduced with miR-509 lentivirus or empty vector (EV#1) at MOI  = 2, and transduced GFP^+^ cells were mixed with an equal number of mock-transduced cells (GFP^–^) 7 days later to achieve an initial culture of ∼50%GFP^+^ cells; this was designated Day 0 and the %GFP^+^ cells (pre-gated on viable cells) was assessed weekly by flow cytometry. Means ± SEMs are shown for 3 independent experiments. (C) Enforced expression of mature miR-509-5p and miR-509-3p NALM6 cells, as assayed by qRT-PCR. NALM6 cells were transduced with miR-509 lentivirus to MOI  = 2, and total RNA was collected at 7 days after transduction. U18 was used as the loading control, and normalized to EV#1-transduced NALM6 cells. Means ± SEMs of 3 independent experiments. (D) Expression of mature miR-509-5p was determined by miR microarray analysis in B-ALL, T-ALL and AML cell lines and primary samples, B cells, CD34^+^ HSPCs, granulocytes, monocytes and T cells. Dotted line represents normalized microarray intensity of 2 whereby any value <2 denotes undetectable expression. Data points shown are means ± SEMs. Expression data is accessible through GEO Series accession number GSE51908 [32]. (E) Expression of mature miR-509-3p and miR-18a as determined by miR microarray analysis similar to (D). (D, E) Data shown for miR-18a is only for the NALM6 cell line.

We applied our functional miR-HTS to screen a pooled lentivirus library of 578 human miRs or miR clusters for their growth-regulatory properties in human NALM6 B-ALL cells and identified candidate miRs as previously described [26]. 4 miRs (miR-381, miR-509, miR-550a, and miR-873) and 1 miR cluster (miR-432∼136) inhibited NALM6 growth in at least 2 of 3 replicate screens performed. In order to confirm the growth inhibitory effects of the candidate miRs identified from the functional screen, each of the 5 miR or miR cluster candidates was cloned into a lentiviral expression vector downstream of green fluorescent protein (GFP) (Figure 1A). We expressed the miR-432∼136 cluster as a single unit rather than as 2 individual miRs, to recapitulate the way they were screened and because the 2 miRs may cooperate. The growth inhibitory potential of each candidate miR or miR cluster was then tested, by performing multiple GFP competition assays [33], [34]. NALM6 cells were transduced with each of the 5 miR lentiviruses (>80% GFP^+^ cells), and each culture was then mixed with GFP^–^ cells to obtain an initial culture with ∼50% GFP^+^ cells. If enforced expression of a given miR or miR cluster inhibited NALM6 growth, the %GFP^+^ cells in culture would decrease over time. For NALM6 cells transduced with the control empty vector, the %GFP^+^ cells remained stable at ∼50% over the 5-week GFP competition assay (Figure 1B). Similarly, no change in %GFP^+^ cells was observed over 35 days in the GFP competition assays for miR-381, miR-550a, miR-873 and miR-432∼136 (Figure S1A-S1D). In contrast, NALM6 cells transduced with miR-509 lentivirus were out-grown by the GFP^–^ cells; the %GFP^+^ cells decreased from 46% at assay day 0 to 10% 35 days later (Figure 1B).

As expected, miR-509-5p and miR-509-3p were strongly overexpressed in miR-509-transduced NALM6 cells as assayed by qRT-PCR (Figure 1C). Similarly, overexpression of miR-381, miR-550a, miR-873, and miR-432 was achieved by lentiviral transduction (Figure S1E). These results indicate that miR-381, miR-432, miR-550a, and miR-873 do not inhibit growth of NALM6. However, no expression of miR-136 was detected in miR-432∼136 cluster-transduced NALM6 cells. This lack of miR-136 expression could be due to lack of necessary cis-regulatory elements or trans-regulatory factors required for miR-136 biogenesis; we did not investigate the possibility that an alternative approach to successfully express miR-136 in NALM6 would validate a growth inhibitory role for this miR. Instead, we decided to focus on miR-509 for further studies.

Our miR microarray expression analyses [32] (GEO Series accession number GSE51908) revealed undetectable endogenous levels of mature miR-509-5p and miR-509-3p in NALM6 and other acute leukemia cell lines (Figure 1D, 1E), as well as in primary leukemia cases and CD34^+^ hematopoietic stem-progenitor cells (HSPCs) and blood cell types from normal human donors (Figure 1D, 1E). In absolute qRT-PCR quantifications [30], [31], miR-509-transduced NALM6 cells expressed 1,814±95 copies (mean ± SEM) per cell of miR-509-5p (Table 1), comparable to levels of miR-18a, which for reference is expressed at the 70th percentile of all miRs in NALM6 cells based on our miR microarray data (Figure 1D). MiR-509-3p was expressed at 3,656±117 copies per cell in miR-509-transduced NALM6 cells, also within the physiological range of miR copy numbers per cell (range: <10 to>30,000 copies per mammalian cell) [30].

**Table 1:** Absolute copy number of mature miR-509 and miR-18a RNA per NALM6 cell.

	Copy number per NALM6 cell transduced with	
qRT-PCR assay	Empty vector #1	miR-509
**miR-509-5p**	<10	1,814±95
**miR-509-3p**	<10	3,656±117
**miR-18a**	1,591±105	1,415±53

RNA was isolated from NALM6 cells on day 7 after transduction with either control empty vector #1 or miR-509, and absolute qRT-PCR quantification was performed for miR-509-5p, miR-509-3p or miR-18a. Copy number per cell was estimated based on standard curves of miR-509-5p, miR-509-3p or miR-18a using DNA oligonucleotides. For reverse transcription, 10 ng RNA (equivalent to 800 cells, i.e. 12.5 pg of total RNA per cell) was used in each reaction. Means ± SEMs of 3 independent experiments.

### MiR-509 reduced NALM6 cell growth by 2 additional independent assays

**Figure 2:**
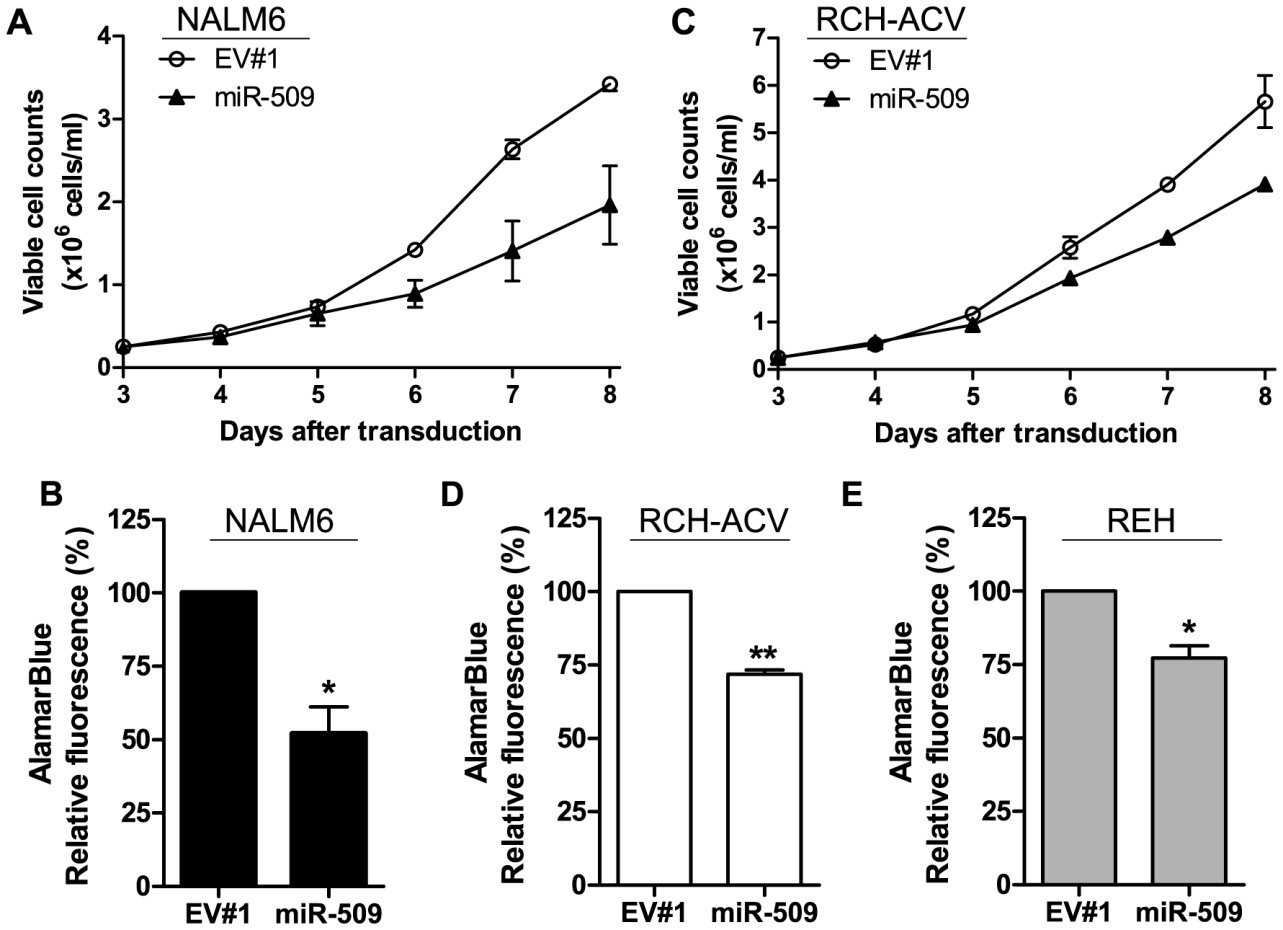
Enforced miR-509 resulted in inhibition of growth of 3 B-ALL cell lines, NALM6, REH and RCH-ACV. (A) Viable cell numbers measured via trypan blue dye exclusion counts of NALM6 cells transduced with either miR-509 lentivirus or empty vector (EV#1); 25,000 cells were plated for each sample starting at 3 days after transduction. (B) AlamarBlue cell growth assay on day 7 after transduction of NALM6 cells transduced with either miR-509 lentivirus or EV#1. Values for miR-509 were normalized to EV#1. (C) Viable cell counts of RCH-ACV cells based on trypan blue exclusion counts, initial plating of 25,000 cells for both samples on 3 days after transduction. Means ± SEMs are plotted, and SEMs for miR-509 were very small. (D) Cell growth of RCH-ACV transduced with either EV#1 or miR-509 overexpressing lentivirus using alamarBlue cell growth assay conducted on day 7 after transduction. Values for miR-509 were normalized to EV#1. (E) MiR-509-transduced REH cells reduced growth compared to EV#1 in an alamarBlue cell growth assay. Cells were transduced 7 days prior to addition of alamarBlue. (A to E) Means ± SEMs, 3 independent experiments done in triplicates. Statistical analysis was done by Student's *t* test. *p<0.05, **p<0.01.

To further confirm the effect of miR-509 on NALM6 cell growth, we performed trypan blue dye exclusion cell counts and alamarBlue assays. At 8 days after transduction, cultures of miR-509-transduced NALM6 cells contained 43% fewer viable cells than empty vector-transduced cells by trypan blue counts (Figure 2A). Similarly, miR-509-transduced NALM6 cells had 48% reduced (p<0.05) cell growth, as compared to empty vector-transduced cells using the alamarBlue assay (Figure 2B). Since the alamarBlue dye-based assay measures the reducing environment within cells, which is linked to mitochondria metabolism [35], we examined whether miR-509 affected mitochondrial membrane potential. No difference in mitochondrial membrane potential was observed between miR-509-transduced and empty vector-transduced NALM6 cells (data not shown).

### MiR-509 inhibited growth of RCH-ACV and REH B-ALL cell lines

We next examined if the growth inhibitory effects of miR-509 extended to other B-ALL (RCH-ACV and REH), T-cell ALL (T-ALL; Jurkat and KARPAS-45) or myeloid leukemia (K562) cell lines. MiR-509-transduced RCH-ACV cells had ∼30% reduced growth by trypan blue on day 8 after transduction or alamarBlue assay on day 7 after transduction (Figure 2C, 2D). In addition, miR-509-transduced REH cells had 23% (p<0.05) reduced growth in the alamarBlue assay (Figure 2E). In contrast, no reduction in cell growth was observed in Jurkat, KARPAS-45 or K562 cells transduced with miR-509 as compared to control empty vector using alamarBlue assays (Figure S2A–S2D). This was despite documented overexpression of miR-509 in these transduced cell lines (Figure S3). Thus, miR-509 inhibited the growth of all 3 tested human B-ALL cell lines, NALM6, RCH-ACV and REH.

### MiR-509-transduced NALM6 cells had a lower proportion of cells in cell cycle S-phase and increased apoptosis

**Figure 3:**
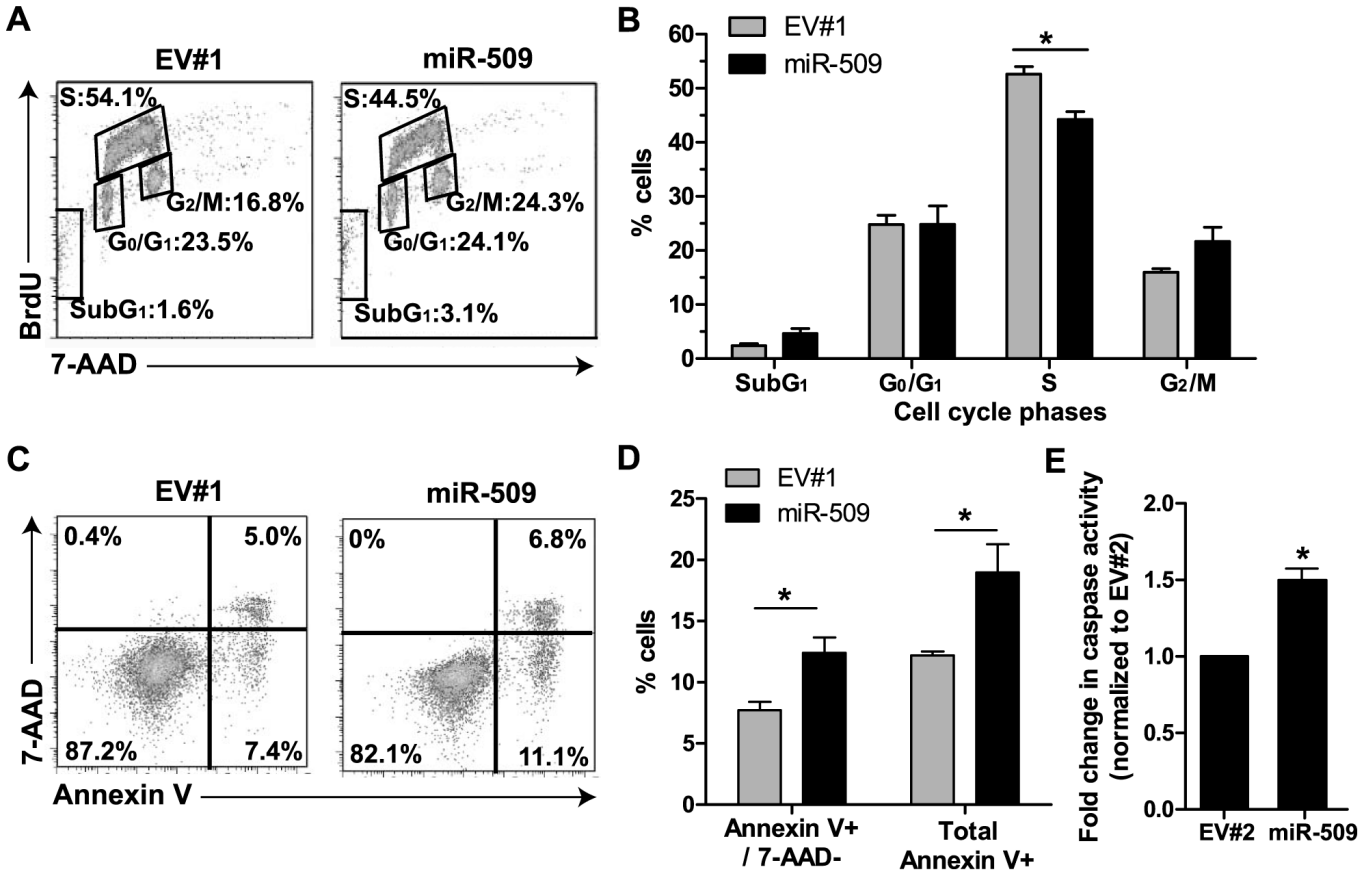
Enforced miR-509 expression in decreased proportion of cells in S-phase, induced apoptosis and activated caspase-3/7. (A) Representative flow cytometric plots showing cell cycle distribution of NALM6 cells transduced with empty vector (EV#1) or miR-509 overexpressing lentivirus. On day 3 after transduction, cells were labeled with BrdU for 1 h. Cells were then fixed overnight and stained on the next day with both BrdU and 7-AAD before analysis by flow cytometry. Percent of cells at each phase of cell cycle are boxed as indicated. (B) Frequencies of cells at the different phases of cell cycle. Means ± SEMs of 3 independent experiments with statistical analysis by Student's *t* test. *p*<*0.05. (C) Representative flow cytometric plots of cell death distribution of NALM6 cells transduced with EV#1 or miR-509 overexpressing lentivirus. Cells were stained with both Annexin V and 7-AAD before analysis by flow cytometry on day 4 after transduction. Numbers represent the frequency in each quadrant. (D) Frequencies of apoptotic cells which are Annexin V-positive (Annexin V^+^) and 7-AAD negative (7-AAD^–^), as well as total Annexin V^+^. Means ± SEMs of 3 independent experiments with statistical analysis by Student's *t* test. *p*<*0.05. (E) NALM6 cells were transduced with empty vector #2 (EV#2) or miR-509 overexpressing lentivirus, and cells were seeded in 384-well plate on day 3 after transduction. On day 7 after transduction, caspase-3/7 activity was measured and fold-change in caspase activity was normalized to EV#2. Means ± SEMs of 3 independent experiments done in triplicates, with statistical analysis by Student's *t* test. *p*<*0.05.

To investigate the cellular mechanisms by which enforced miR-509 expression inhibits growth, we examined whether miR-509 regulates cell cycle progression by conducting BrdU/7-AAD staining [36]. 4 days after transduction, miR-509-transduced NALM6 had fewer cells in S-phase than empty vector-transduced cells (Figure 3A), and this was statistically significant (Figure 3B, p<0.05). In addition, there were slightly elevated numbers of cells in the subG_1_ and G_2_/M phases, but these differences were not statistically significant. To investigate if miR-509 promotes cell death via apoptosis, Annexin V/7-AAD staining was performed. 4 days after transduction, miR-509-transduced NALM6 cells had 1.5-fold (p<0.05) higher numbers of Annexin V^+^/7-AAD^–^ apoptotic cells and 1.4-fold higher numbers of Annexin V^+^ dying/dead cells (p<0.05), as compared to empty vector-transduced cells (Figure 3C, 3D). Consistent with these findings, we detected a 1.5-fold increase (p<0.05) in activated capase-3/7 activity in miR-509-transduced NALM6 cells as compared to empty vector-transduced cells (Figure 3E).

### Informatics prediction of RAB5C as a target of miR-509

**Figure 4:**
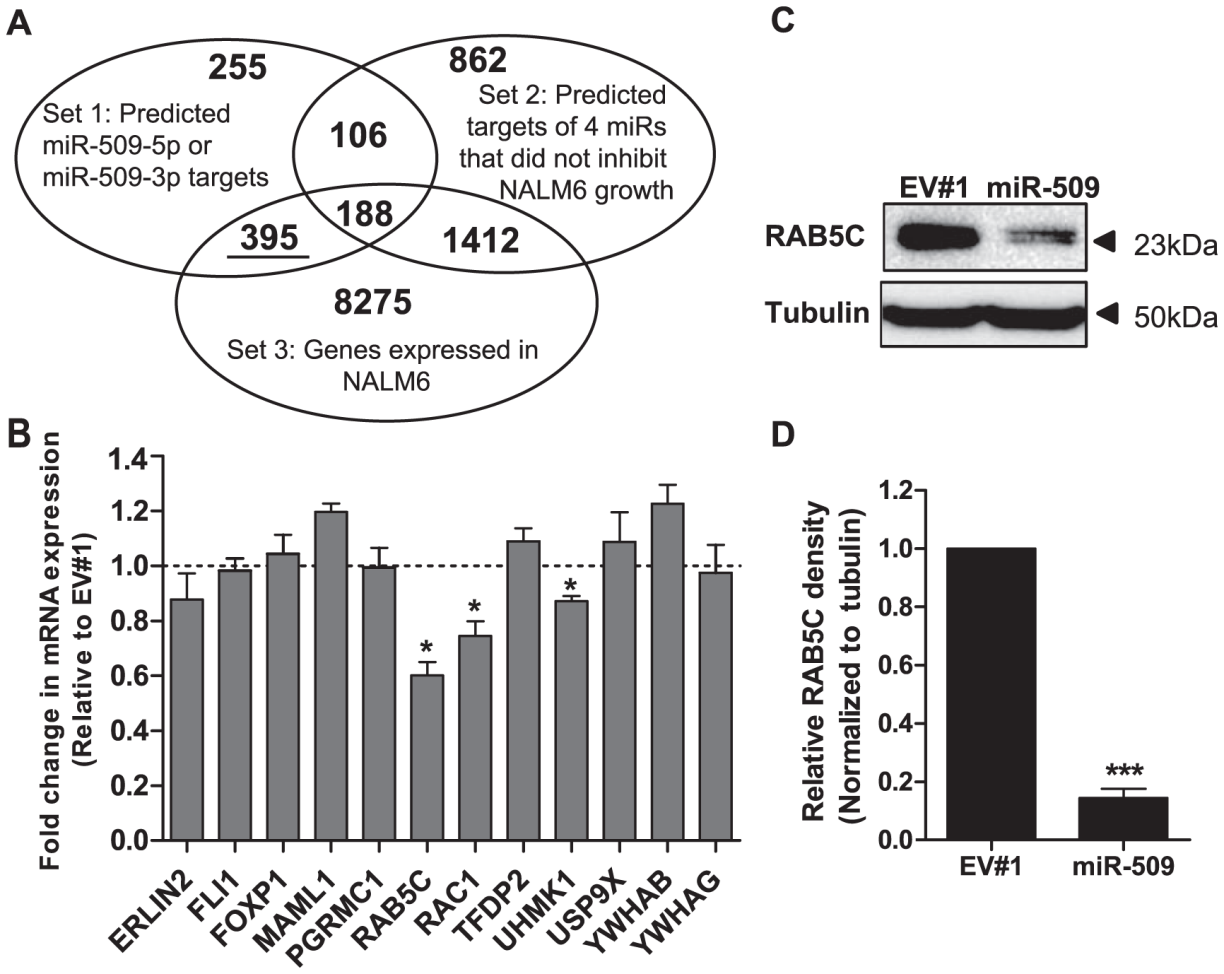
Identifying mRNA targets of miR-509. (A) Venn diagram showing the number of mRNAs that do not overlap, or are shared between each set in our *in silico* strategy to identify relevant targets of miR-509. Set 1 refers to the list of predicted targets of miR-509-5p or miR-509-3p from TargetScan6.2 or miRDB. Set 2 is the list of predicted targets of miRs tested to not inhibit NALM6 growth (i.e. miR-550a, miR-873, miR-381 and miR-432) from TargetScan6.2 or miRDB, while Set 3 is the list of mRNA that is expressed in NALM6, as determined by genome-wide microarray profiling downloaded from the Cancer Cell Line Encyclopedia and its expression levels are denoted in the microarray dataset as “marginal” or “present”. (B) Expression levels of 12 putative targets of miR-509 as determined by qRT-PCR. RNA was isolated from NALM6 cells transduced with EV#1 or miR-509 overexpressing lentivirus at 7 days after transduction. All values were normalized to GAPDH and fold-change was calculated relative to EV#1 sample. Data represents means ± SEMs of 3 independent experiments, with statistical analysis by Student's *t* test. *p*<*0.05. (C) Representative western blots of RAB5C expression. NALM6 cells were transduced with either EV#1or miR-509 overexpressing lentivirus, and whole cell lysates were harvested at 7 days after transduction. α-tubulin was used for loading control. (D) Densitometry analysis of RAB5C expression of western blot in (C) and 2 other independent experiments. α-tubulin was used for normalization, and relative densitometry was then calculated compared to EV#1. Data shown represent means ± SEMs, with statistical analysis by Student's *t* test. ***p<0.001.

To identify targets of miR-509 that might mediate growth of B-ALL cells, we used a filtering strategy to prioritize the many predicted targets of miR-509 (Figure 4A). First, we downloaded the sets of predicted mRNA targets of miR-509-5p and miR-509-3p (Set 1), as well as those of the 4 miRs that we had shown not to inhibit NALM6 growth (i.e. miR-381, miR-432, miR-550a and miR-873; Set 2) from the TargetScan6.2 [37] and/or miRDB [38], [39] miR target prediction databases. Since NALM6 cells transduced with miR-432∼136 did not result in miR-136 overexpression, we did not include miR-136 targets in Set 2 (Figure 4A). Next, we downloaded the gene expression profile of NALM6, determined by genome-wide microarray profiling as listed in the Cancer Cell Line Encyclopedia (CCLE) [40] and focused on genes which have detectable expression in NALM6 (i.e. annotated as “marginal” or “present” in CCLE; Set 3). Then, we intersected these 3 sets of mRNAs [41] to identify the subset of genes expressed in NALM6 and predictively targeted by miR-509, but not predictively targeted by the 4 miRs that did not inhibit NALM6 growth. This resulted in a set of 395 genes (listed in Table S6). This list was subsequently reduced to 74 genes by selecting for genes known to participate in growth regulation based on annotations at NCBI's “Gene” database, DAVID bioinformatics resources [42], [43], as well as our own literature searches. Of these 74 predicted targets of miR-509, 12 genes previously demonstrated in the literature to be either involved in leukemia and oncogenesis (ERLIN2, FLI1, FOXP1, MAML1, RAC1, YWHAB and YWHAG), or predicted as miR-509 targets by both TargetScan6.2 and miRDB (PGRMC1, RAB5C, RAC1, TFDP2, UHMK1, USP9X) were selected for initial qRT-PCR analysis. We used this informatic filtering strategy, as compared to performing global differential gene expression analysis such as microarray analysis, to enable us to rapidly and at low cost identify target genes-of-interest. 3 of these 12 predicted targets (RAB5C, RAC1, and UHMK1) were down-regulated by miR-509 at the mRNA level (Figure 4B). RAB5C mRNA levels showed the greatest reduction, with a 40% lower level (p<0.05) in miR-509-transduced than in empty vector-transduced NALM6 cells (Figure 4B). Correspondingly, RAB5C protein was 85% (p<0.001) lower in miR-509-transduced cells by western blotting (Figure 4C, 4D). We also observed a ≥86% decrease in RAB5C protein levels in miR-509-transduced RCH-ACV and REH cells as compared to empty vector (Figure S4). Since RAB5 has been implicated in cell cycling [44], [45] and is one of the top 3 predicted targets of miR-509-3p by both TargetScan6.2 (Total context+ score  = −0.65) and miRDB (Target score  = 91), we focused our subsequent studies on RAB5C.

### MiR-509 directly targets RAB5C

**Figure 5:**
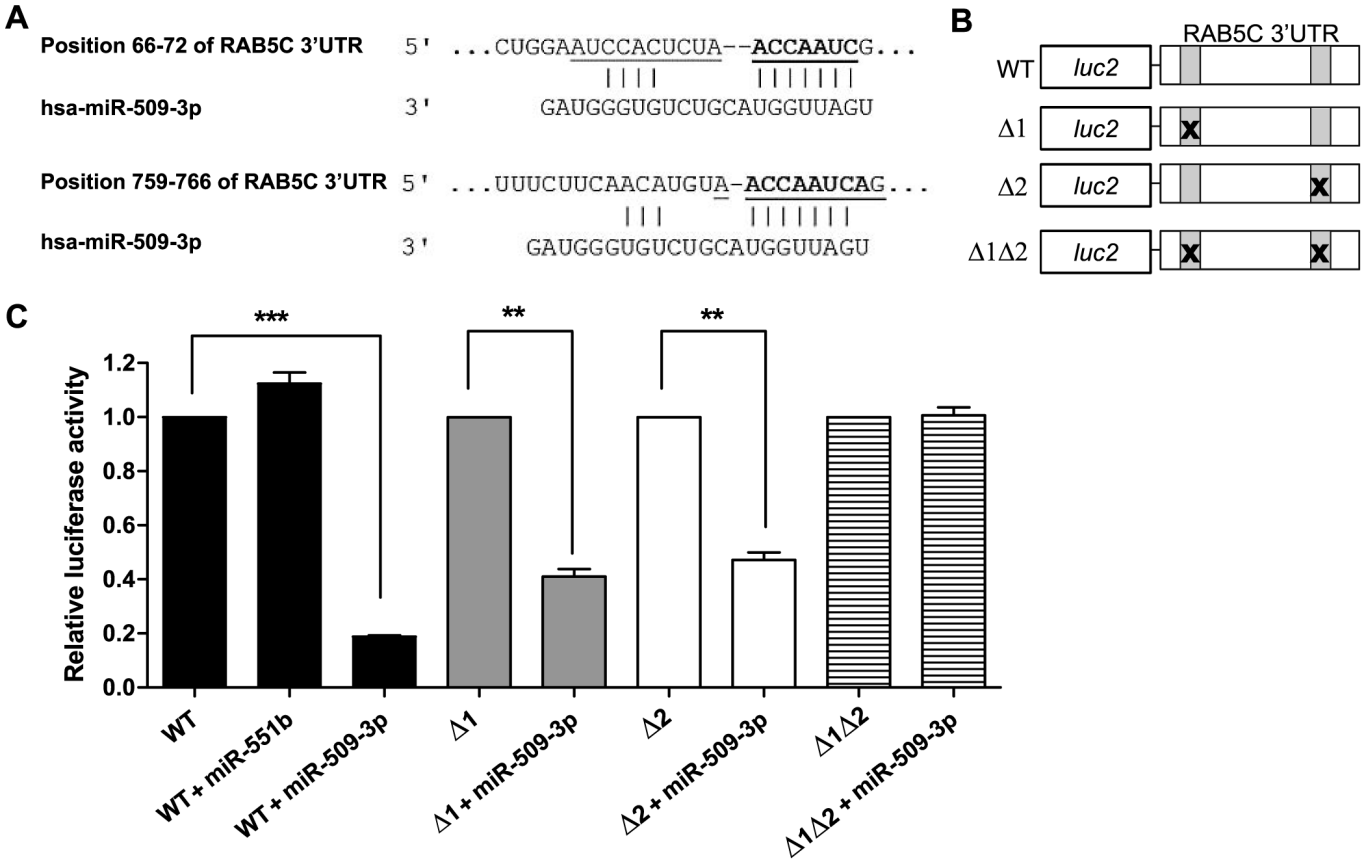
RAB5C is a direct target of miR-509. (A) Sequence alignment of RAB5C to miR-509-3p predicted by TargetScan6.2. The full length 3′UTR of RAB5C is 803 bases. Sequences shown in bold refer to position 66–72 and 759–766 of RAB5C 3′UTR where miR-509-3p is predicted to target. The underlined sequences were deleted in the RAB5C-3′UTR deletion constructs listed in (B) for the luciferase assay. (B) Schematic representation of luciferase vector constructs used in luciferase assay. Full length RAB5C 3′UTR was cloned downstream of the firefly luciferase gene (luc2) in the pmirGLO luciferase vector. Wild type RAB5C 3′UTR is listed as WT. Grey boxes indicate the 2 predicted miR-509-3p target sites (66–72 and 759–766), and the “X” indicates the deletion sites present in the deletion (Δ) constructs. (C) Luciferase assay demonstrates that RAB5C 3′UTR is targeted by miR-509-3p via 2 binding sites. 293T cells were transfected with the 300ng of the indicated luciferase plasmids and 50nM of miR mimics, and harvested for luciferase assay 48 h after transfection. All values were first normalized to Renilla luciferase. Relative luciferase activity was then calculated by normalizing co-transfection of miR mimics plus luciferase constructs to cells transfected with only the respective luciferase construct. MiR-551b was used as a non-targeting miR negative control. Data shown represent means ± SEMs of 3 independent experiments, with statistical analysis by Student's *t* test. **p<0.01, ***p<0.001.

To examine if miR-509 directly represses RAB5C, we employed RAB5C-3′UTR luciferase reporter assays. There are 2 miR-509-3p binding sequences in the 3′UTR of RAB5C (Figure 5A), as predicted by both miRDB and TargetScan6.2. Both miR-509-3p binding sequences are present in the RAB5C 3′UTR of several species including human, mouse, rat, horse and dog, suggesting that the regulation of RAB5C by miR-509 is also conserved. We cloned the full-length wild type (WT) 3′UTR of RAB5C downstream of firefly luciferase gene (*luc2*) in the pmirGLO luciferase vector and also generated 3 luciferase constructs containing 1 (Δ1 or Δ2) or both (Δ1Δ2) deletions of miR-509-3p binding sites (Figure 5B). Co-transfection of miR-509-3p mimic and RAB5C-3′UTR WT luciferase vector resulted in 81% lower (p<0.001) relative luciferase activity than in cells transfected with RAB5C-3′UTR WT luciferase vector alone (Figure 5C). Co-transfection of the non-targeting miR-551b mimic plus the RAB5C-3′UTR WT luciferase vector did not repress luciferase activity. Co-transfection of either RAB5C-3′UTR-luciferase deletion construct, Δ1 or Δ2, plus miR-509-3p mimic resulted in >50% lower (p<0.01) relative luciferase activity than cells transfected with only the indicated RAB5C-3′UTR deletion constructs. Co-transfection of Δ1Δ2 construct (in which both predicted miR-509-3p binding sites were deleted) with miR-509-3p mimic abolished the reduction in luciferase signal. This indicated that miR-509 directly targets the 3′UTR of RAB5C via both predicted miR-509-3p binding sites.

### RAB5C mediates the growth-inhibitory effect of miR-509

**Figure 6:**
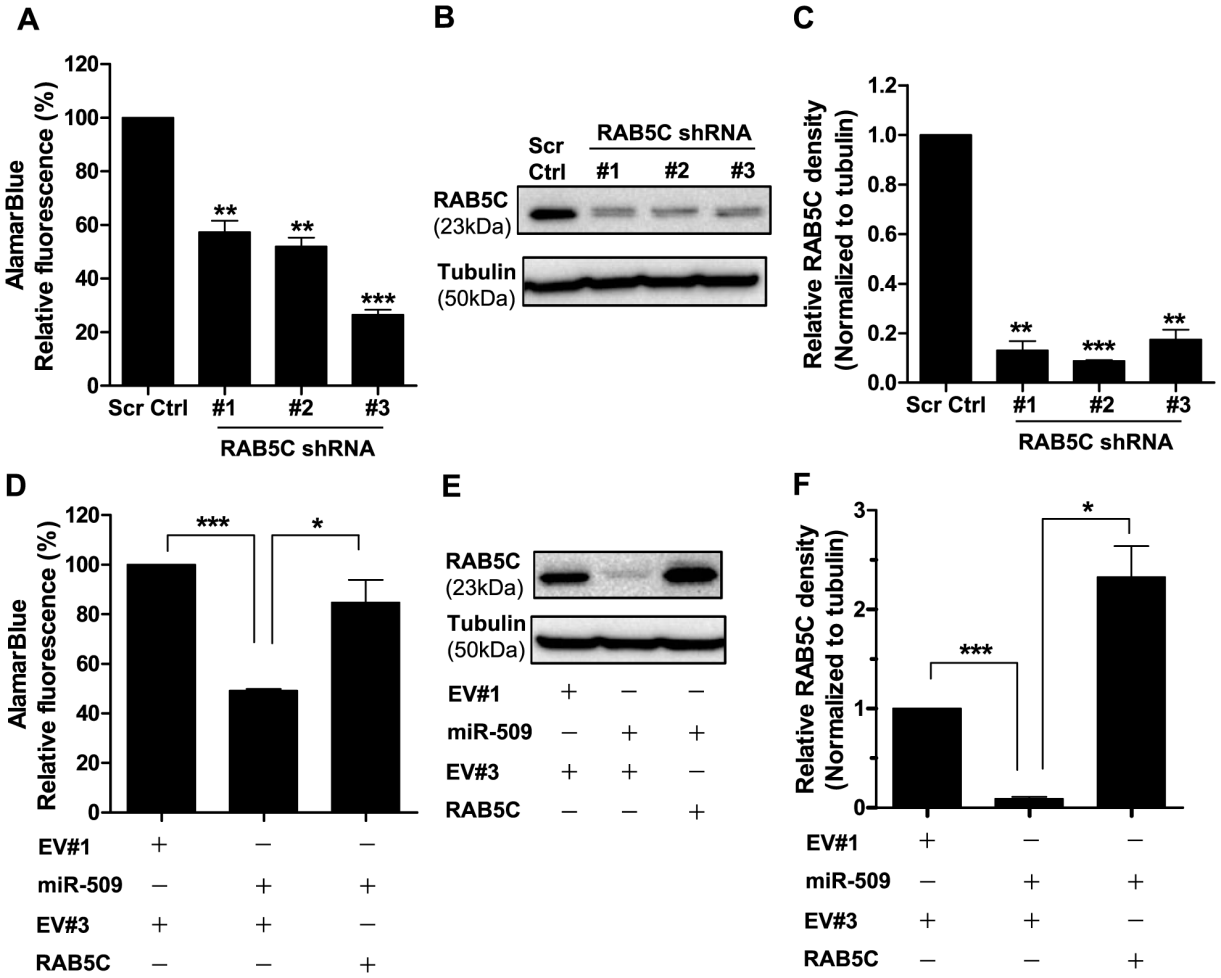
RAB5C mediates the growth-inhibitory effect of miR-509. (A) AlamarBlue assay of NALM6 cells transduced with either lentivirus of scrambled control (Scr Ctrl), shRNA#1, shRNA#2 or shRNA#3 for RAB5C on 7 days after transduction. Data represent means ± SEMs of 3 independent experiments with statistical analysis by Student's *t* test. **p<0.01 and ***p<0.001. (B) Representative western blot of NALM6 cells transduced with lentivirus of scrambled control (Scr Ctrl), shRNA#1, shRNA#2 or shRNA#3 for RAB5C. Protein lysates were harvested 7 days after transduction and α-tubulin was used as a loading control. (C) Quantitation of western blots shown in (B) and 2 other independent experiments. Relative densitometry values were calculated relative to Scr Ctrl. Results show means ± SEMs with statistical analysis by Student's *t* test. **p<0.01 and ***p<0.001. (D) Enforced expression of RAB5C without its 3′UTR rescues miR-509-mediated growth inhibition. NALM6 cells were co-transduced with the indicated plasmids, and alamarBlue assay was read at 7 days after transduction. The empty lentiviral vector in this experiment is EV#3, which does not have RAB5C cloned in. Results show means ± SEMs of 3 independent experiments, with statistical analysis using Student's *t* test. *p<0.05 and ***p<0.001. (E) Representative western blot of NALM6 cells co-transduced with lentivirus of the indicated plasmids. Protein lysates were harvested 7 days after transduction and α-tubulin was used as a loading control. (F) Quantitation of western blots shown in (E) and 2 other independent experiments. Relative densitometry values were calculated relative to EV#1 and EV#3. Results show means ± SEMs with statistical analysis by Student's *t* test. *p<0.05 and ***p<0.001.

We then examined if reduced RAB5C is responsible for the functional effects of miR-509. To determine if repression of RAB5C would phenocopy the growth suppressive effect of miR-509, NALM6 cells were transduced with 3 different lentiviruses, each containing a distinct shRNA against RAB5C. In alamarBlue assays, all 3 shRNAs inhibited NALM6 cell growth by ≥42% (p<0.01) as compared to cells transduced with the scrambled control (Figure 6A). We verified that all 3 shRNAs resulted in ≥80% decreased RAB5C protein levels (p<0.01) in NALM6 cells (Figure 6B, 6C) using western blotting.

In order to determine whether RAB5C mediates miR-509 induced growth inhibition in NALM6 cells, we performed a rescue experiment. We cloned the RAB5C open reading frame (ORF) without its 3′UTR into a lentiviral vector. In alamarBlue assays, NALM6 cells co-transduced with miR-509 plus empty vector had 51% lower growth (p<0.001) than cells co-transduced with the 2 control empty lentiviral vectors (Figure 6D). In contrast, NALM6 cells co-transduced with miR-509 plus RAB5C lentiviruses had 36% greater growth than cells co-transduced with miR-509 plus the empty vector (p<0.05). Overexpression of RAB5C ORF in NALM6 cells co-transduced with miR-509 was confirmed by western blotting (Figure 6E, 6F). Thus, RAB5C rescued, in large part, the growth inhibitory effects of miR-509.

## Discussion

In this study, we conducted a functional miR-HTS in NALM6 cells and identified miR-509 as a novel inhibitor of human B-ALL cell growth. Using NALM6 B-ALL cell line, enforced expression of miR-509 reduced NALM6 B-ALL cell growth in 3 independent growth assays. MiR-509-mediated growth inhibition was also observed in 2 additional B-ALL cell lines, REH and RCH-ACV. However, miR-509 is not a global inhibitor of cell growth, as enforced miR-509 expression in 2 T-ALL (Jurkat and KARPAS-45) and 1 myeloid leukemia (K562) cell lines did not inhibit growth. Susceptibility to miR-509 growth inhibition is likely due to differential expression or differential dependence upon miR-509 target genes for cell proliferation and survival [46]. More extensive testing will be necessary to determine if the growth inhibitory effects of miR-509 might be specific to B-ALL cells or some molecularly-defined subset of leukemias or shared with other cancer types. MiR-509 has been reported previously to be down-regulated in renal cell carcinoma as compared to normal tissue counterparts [47], [48]. Since miR-509-5p and miR-509-3p are undetectable in normal or leukemic hematopoietic cells, miR-509 does not qualify as a tumor suppressor miR for leukemias. The lack of miR-509 expression in healthy donor blood cell types and CD34^+^ HSPCs [32] exemplifies the importance of functional screening to identify growth-suppressing miRs, as expression profiling comparing acute leukemia cases versus healthy donor samples would not have identified miR-509 as a miR capable of inhibiting leukemia cell growth.

We further observed that enforced miR-509 expression reduced the number of actively proliferating cells and increased apoptotic and dead NALM6 cells, indicating that miR-509 reduces cell proliferation and survival. Our observations in NALM6 are consistent with previous reports that both miR-509-5p [47] and miR-509-3p [48] suppressed cell growth and induced apoptosis in a human renal cancer cell line.

To identify relevant miR-509 targets, we may in the future employ biochemical or genomic techniques [49], [50] to identify all of the targets of miR-509 in NALM6 cells. In the initial study herein, we instead used bioinformatics to define a subset of predicted miR-509 target genes known to be expressed in NALM6 cells but not predicted to be targeted by the miRs that failed to inhibit NALM6 cell growth. We then selected those targets known to be involved in cellular processes that regulate growth (e.g. proliferation, cell cycle, cell death, oncogenes), resulting in a set of 74 growth-related predicted miR-509 targets. Using qRT-PCR to assess levels of 12 of these 74 targets in miR-509-transduced versus empty vector-transduced NALM6 cells, 3 predicted miR-509 targets were reduced in miR-509-transduced NALM6 cells. Although the mRNAs of 9 of the 12 tested predicted miR-509 targets were not reduced in miR-509-transduced NALM6 cells, some of these may still be targets of miR-509 as they might be inhibited at the translational level [51]. However, given that reduction at the mRNA level was observed in ≥84% of miR targets with reduced protein levels [52], we decided to focus in the study herein on predicted targets inhibited by miR-509 at the mRNA level. Moreover, RAB5C mRNA was the target most reduced in response to miR-509 expression, and therefore we focused on RAB5C for further experiments. We showed that miR-509 indeed binds to the 3′UTR of RAB5C via the 2 thermodynamically predicted sites. Upon effective knockdown of RAB5C using each of 3 shRNA constructs, NALM6 cell growth was reduced; thus, RAB5C knockdown phenocopied the growth inhibition observed by enforced miR-509 expression. Our observations that co-transduction of the RAB5C ORF lacking its 3′UTR (therefore no longer regulated by miR-509) rescues miR-509-mediated growth inhibition indicates that reduction of RAB5C is a major mechanism of miR-509-mediated NALM6 growth inhibition. Thus, while future studies may find additional targets of miR-509-5p and/or miR-509-3p that contribute to miR-509-mediated growth inhibition, our current results demonstrate that RAB5C is a novel target of miR-509 and an important driver of the growth of human B-ALL cells.

As members of the Rab family of small monomeric GTPases, RAB5 molecules are central in coordinating vesicle trafficking, particularly in the early stages of endocytosis [53]–[55]. In addition to cell cycling [44], [45], RAB5 has been reported to play a role in other cellular pathways including autophagy [56], [57] and mTOR signaling [58], [59]. In humans, the RAB5 subfamily includes 3 isoforms, which may have distinct functions [60]–[62]. RAB5C isoform has specifically been shown to be involved in cell migration during zebrafish gastrulation [63], cell invasion via regulation of growth factor-stimulated recycling of integrin [64], and cell motility through RAC1 [65]. Protein alignment of RAB5A and RAB5B revealed 83% and 86% sequence similarity to RAB5C protein, respectively [66]. Neither miR-509-5p nor miR-509-3p is predicted to target RAB5A, and we did not detect any change in RAB5A expression in miR-509-transduced NALM6 cells (Figure S5). TargetScan6.2 predicts a miR-509-5p binding site in the 3′UTR of RAB5B (total context+ score  = −0.04). However, using qRT-PCR, we did not detect RAB5B expression in NALM6 cells (Figure S6).

Previously, it has been shown that knockdown of all 3 RAB5 isoforms, but not knockdown of individual isoforms, in human cells resulted in defective alignment of chromosomes, delayed progression though mitosis and defective chromosome segregation [45]. While our BrdU/7-AAD analysis did not detect significantly elevated numbers of miR-509-transduced NALM6 cells in cell cycle phase G_2_/M, this could be due to expression of the compensatory isoform RAB5A. The downstream mechanism by which RAB5C regulates B-ALL cell growth remains unclear. Given that RAB5 is a key regulator of the endosome pathway, the impaired cell growth in miR-509-transduced or RAB5C-knockdown cells might be due to aberrant recycling of surface growth receptors, such as transferrin receptor [53]. In HeLa cells, knockdown of all 3 RAB5 isoforms resulted in delayed internalization of transferrin receptor and reduced uptake of transferrin [62]. Since the transferrin receptor is important in regulation of intracellular iron concentration which in turn affects cell proliferation [67], [68], efforts to examine the effects of RAB5C on transferrin receptors and/or other growth-related receptors required for B-ALL growth are ongoing.

Our data indicate that RAB5C is important for B-ALL cell growth. Therefore, we might expect RAB5C to be overexpressed in B-ALL cells as compared to normal counterpart cells. Thus, we queried RAB5C expression in leukemia using the Oncomine cancer microarray database [69], comparing specifically the ‘cancer versus normal’ analysis with a threshold of p-value ≤0.001 and 1.5-fold over-expression. Using these criteria to assess the 14 ‘cancer versus normal’ datasets and focusing solely on leukemia in Oncomine, RAB5C was overexpressed in the dataset of B-ALL patient samples harboring the t(12;21) chromosomal translocation (producing the TEL/AML-1 fusion protein oncogene) as compared to normal B-lymphoid precursors (pro/pre–B cells and immature B cells) from healthy donors [70]. In this TEL/AML-1 B-ALL subset, there was 1.8-fold elevated (average; Student's *t* test, p = 3.67^−6^) RAB5C expression (Figure S7). The overexpression of RAB5C in this B-ALL subset, along with our findings that RAB5C supports growth of B-ALL cells, suggests that RAB5C may represent a target for treatment of the TEL/AML-1 B-ALL subset, especially if future studies reveal that growth of primary B-ALL cases harboring TEL/AML-1 is highly dependent on RAB5C. In addition, future work may include determining whether RAB5C overexpression in hematopoietic stem cells can drive B-ALL development.

Despite using the same candidate selection criteria, we were surprised to find that the candidate validation rate (20%) of the miR-HTS conducted in NALM6 cells herein was lower than previously observed for miR-HTS conducted in the IMR90 human lung fibroblast (75% validation rate) [26]. False positives in the miR-HTS may be due to the Monte Carlo effect [71], [72], where low template amounts might result in sporadic amplification at the reference time point sample but not the samples at the later time points. Consequently, such a candidate miR will not be validated. Indeed, 2 of the 4 false-positive candidate miRs in this study were detected only at the first or the first 2 time points. Neither of the other 2 false-positive candidates were detected at any of the 4 times points, including the reference time point. We designated these as candidates because we have used the same batch of lenti-miR library to conduct miR-HTS in a total of 4 cell lines (i.e. IMR90, NALM6 and 2 other cell lines) and both of these lenti-miRs were detected at the reference time point in at least one of these 4 cell lines. This suggested to us that the lenti-miR library indeed contained these 2 miR lentiviruses and should have infected the NALM6 as effectively as the other cell lines. Thus, lack of detecting cells containing these 2 lenti-miRs in NALM6 even at the reference time point might be due to a very strong growth-inhibitory effect of these 2 miR candidates. Hence, these 2 miR candidates were included in our validation analyses. However, it is possible that the lenti-miR viruses for these 2 false-positive miR candidates did not actually infect NALM6 cells.

In summary, our findings demonstrate the ability of our miR-HTS platform to identify leukemia cell growth inhibitory miRs and their molecular targets. Our observation that enforced miR-509 expression inhibits growth of B-ALL cell lines provided the clue to identifying the role of RAB5C in the growth of B-ALL cells. To our knowledge, this is the first report of RAB5C as a regulator of B-ALL cell growth. Elucidating the downstream mechanistic roles of RAB5C in growth of human B-ALL cells might suggest novel therapeutic strategies against B-ALL.

## Supporting Information

Figure S1 **No growth defects were observed for 4 other miR candidates using the GFP competition assay.** NALM6 cells were individually transduced with lentivirus of (A) miR-381; (B) miR-550a; (C) miR-873 and (D) miR-432∼136 and empty vector (EV#1) to MOI  = 2. At 7 days after transduction, cells were mixed with mock-transduced cells to 50% GFP^+^ cells and this was set as Day 0. The %GFP^+^ cells (pre-gated on viable cells) of each culture were assessed weekly by flow cytometry for 35 days. Means ± SEMs are shown for three independent experiments. (E) Overexpression of miR candidates in NALM6 cells, as assayed by qRT-PCR. NALM6 cells were transduced with each miR lentivirus to MOI  = 2, and total RNA was collected at 7 days after transduction. U18 was used as the loading control. Values shown were calculated as fold overexpression relative to EV#1-transduced NALM6 cells (EV#1). Means ± SEMs are shown for 3 independent experiments.
(TIF)

Figure S2 **MiR-509 does not regulate the growth of Jurkat, KARPAS-45 and K562 cells.** AlamarBlue cell growth assay was then performed on day 7 after transduction of (A) Jurkat, (B) KARPAS-45 and (C) K562 cells with either miR-509 lentivirus or EV#1. Each cell line was transduced with the indicated lentivirus to MOI  = 2. On day 3 after transduction, cells were seeded at the indicated numbers per well/100 µl media: Jurkat (5×10^3^ cells), KARPAS-45 (3×10^3^ cells) and K562 (1.25×10^3^ cells) in triplicates in 96-well plates. Values for miR-509 were normalized to EV#1. Means ± SEMs, *ns*  =  no statistical significance was detected by Student's *t* test.
(TIF)

Figure S3 **Enforced expression of miR-509 was detected by qRT-PCR in selected T-ALL and myeloid leukemia cell lines transduced with miR-509 lentivirus.** (A) Jurkat, (B) KARPAS-45 and (C) K562 cells were transduced with miR-509 lentivirus or EV#1. On day 7 after transduction, cells were collected for RNA isolation. U18 was used as the endogenous control. Values shown were calculated as fold overexpression relative to each EV#1-transduced cells. Means ± SEMs are shown for 3 independent experiments.
(TIF)

Figure S4 **RAB5C protein levels were decreased in RCH-ACV and REH cells with enforced miR-509 expression.** Representative western blot of RAB5C expression in (A) RCH-ACV and (B) REH. Cells were transduced with either EV#1or miR-509 overexpressing lentivirus, and whole cell lysates were harvested at 7 days after transduction. α-tubulin was used for loading control. The bar graph below represents the densitometry analysis of RAB5C expression of 3 independent experiments, normalized to α-tubulin, and relative densitometry was then calculated compared to EV#1. Data shown represent means ± SEMs, with statistical analysis by Student's *t* test. **p<0.01, ***p<0.001.
(TIF)

Figure S5 **RAB5A mRNA levels show no change in miR-509-transduced NALM6 cells.** NALM6 cells were transduced with empty vector #1 (EV#1) to MOI  = 2, and RNA was isolated at day 7 after transduction for qRT-PCR. All values were normalized to GAPDH and fold-change was calculated relative to EV#1 sample. Data represents means ± SEMs of 3 independent experiments, with statistical analysis by Student's *t* test, *ns*  =  no statistical significance was detected by Student's *t* test.
(TIF)

Figure S6 **Expression of RAB5A and RAB5C, but not RAB5B, was detected in NALM6 cells using qRT-PCR.** NALM6 cells were transduced with empty vector #1 (EV#1) to MOI  = 2, and RNA was isolated at day 7 after transduction for qRT-PCR. Ratio to GAPDH (endogenous control) was calculated as 2E[-(RAB5_Ct_ – GAPDH_Ct_)]. Means ± SEMs, *n* = 3 independent experiments. Value above each bar represents the mean.
(TIF)

Figure S7 **Scatter dot plot of RAB5C mRNA expression in B-ALL cells and pre-B lymphocytes based on Oncomine cancer microarray database.** RAB5C expression in leukemia was examined using the Oncomine cancer microarray database by comparing specifically the ‘cancer versus normal’ analysis and setting a threshold of p-value ≤0.001 and 1.5-fold over-expression. 14 ‘cancer versus normal’ datasets were identified and we focused solely on leukemia in Oncomine. RAB5C was overexpressed by 1.8-fold (average; Student's t test, *p* = 3.67^−6^) in the dataset of B-ALL patient samples harboring the t(12;21) chromosomal translocation (producing the TEL/AML-1 fusion protein oncogene; *n* = 17) as compared to normal B-lymphoid precursors (pro/pre–B cells and immature B cells; *n* = 2) from healthy donors [67]. Error bars represent the mean ± SEM.
(TIF)

Table S1 **List of primers used for cloning of miR hairpin with flanking genomic sequences.** PCR products were first cloned into pJET1.2 and subcloned into empty lentiviral vector #1 (EV#1; pWCC52) downstream of GFP. MiR-509 was then subcloned from pWCC52-miR-509 into empty lentiviral vector #2 (EV#2; pWCC72) downstream of DsRed.
(DOCX)

Table S2 **Primers used for PCR of RAB5C-3′UTR and deletion of miR-509-3p binding sites.** Full length RAB5C-3′UTR was cloned into pmirGLO Dual-Luciferase miRNA Target Expression vector (Promega). This plasmid was then used as a template for site-directed mutagenesis to delete the first miR-509-3p binding sites in RAB5C-3′UTR-luciferase deletion construct, Δ1or Δ1Δ2 using primers Del56-72. For the deletion of the second miR-509-3p binding site in RAB5C-3′UTR-luciferase deletion construct, Δ2 or Δ1Δ2, standard PCR was performed using the Del758-767 primers.
(DOCX)

Table S3 **Primers used in cloning of RAB5C lacking its 3′UTR into pWCC61 lentiviral vector (Empty lentiviral vector #3, EV#3).**
(DOCX)

Table S4 **List of primers used for SYBRGreen qRT-PCR.** Primer sequences were obtained from PrimerBank. Fwd: Forward; Rev: Reverse.
(DOCX)

Table S5 **List of TaqMan microRNA assay ID used for qRT-PCR.**
(DOCX)

Table S6 **List of the 395 predicted targets of miR-509-5p and/or miR-509-3p selected based on filtering strategy shown in** **Figure 4A** **.** These targets were subjected to a filtering strategy presented in Fig. 4A and meet the following criteria: (i) They are predicted targets of miR-509-5p and/or miR-509-3p from TargetScan6.2 and/or miRDB. (ii) These targets are not targets of miR-381, miR-550a, miR-873 and miR-432 as predicted by TargetScan6.2 and/or miRDB. (iii) These targets are expressed in NALM6 cells as determined by genome-wide microarray profiling downloaded from the Cancer Cell Line Encyclopedia and its expression levels are denoted in the microarray dataset as “marginal” or “present”.
(DOCX)
