# Supplementary figures and images for: Regulation of RAB5C Is Important for the Growth Inhibitory Effects of MiR-509 in Human Precursor-B Acute Lymphoblastic Leukemia
Source: PLoS One. 2014 Nov 4;9(11):e111777. doi: 10.1371/journal.pone.0111777 (PMC4219775; doi:10.1371/journal.pone.0111777)

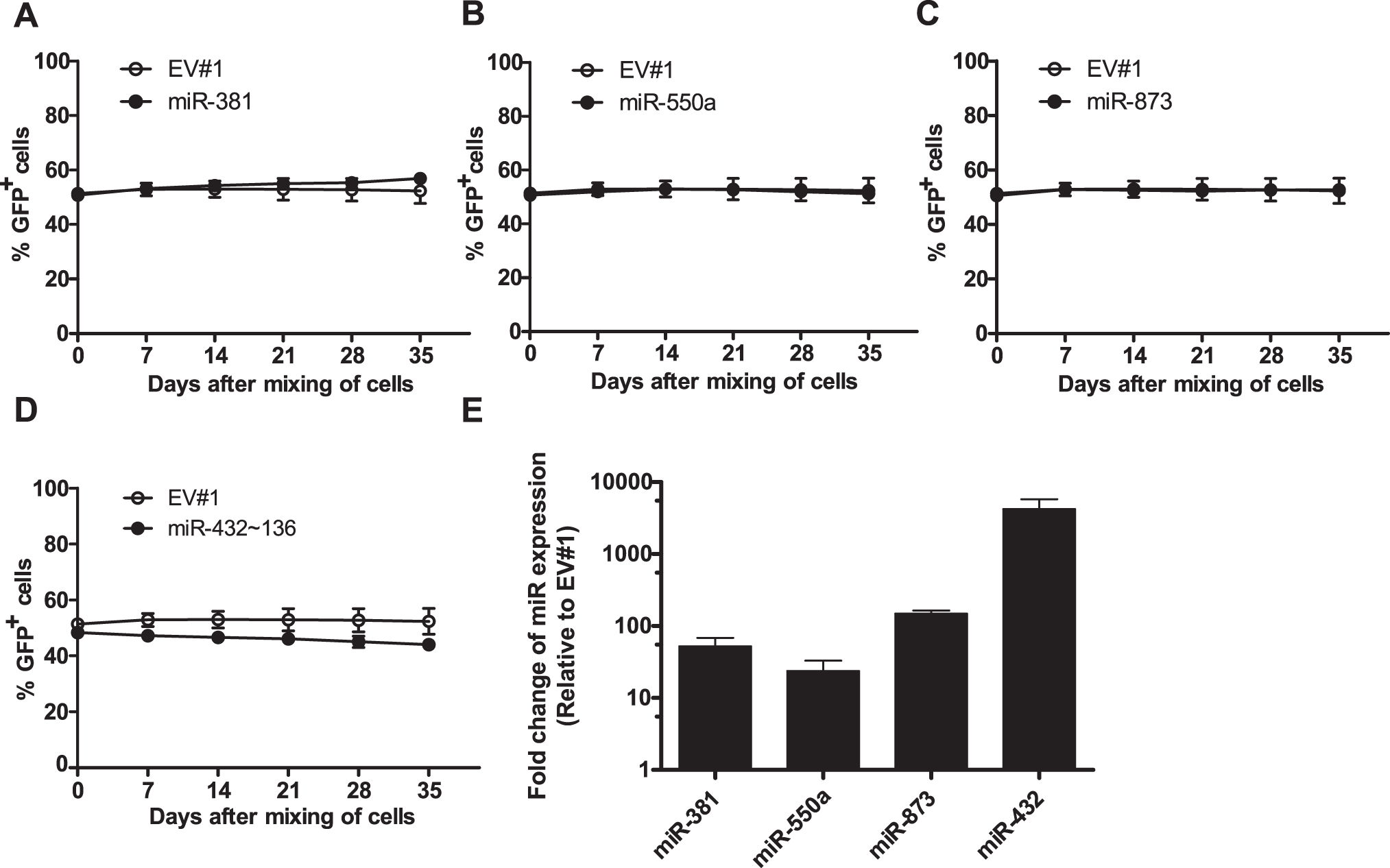

Supplement: Figure S1 — No growth defects were observed for 4 other miR candidates using the GFP competition assay. NALM6 cells were individually transduced with lentivirus of (A) miR-381; (B) miR-550a; (C) miR-873 and (D) miR-432∼136 and empty vector (EV#1) to MOI = 2. At 7 days after transduction, cells were mixed with mock-transduced cells to 50% GFP+ cells and this was set as Day 0. The %GFP+ cells (pre-gated on viable cells) of each culture were assessed weekly by flow cytometry for 35 days. Means ± SEMs are shown for three independent experiments. (E) Overexpression of miR candidates in NALM6 cells, as assayed by qRT-PCR. NALM6 cells were transduced with each miR lentivirus to MOI = 2, and total RNA was collected at 7 days after transduction. U18 was used as the loading control. Values shown were calculated as fold overexpression relative to EV#1-transduced NALM6 cells (EV#1). Means ± SEMs are shown for 3 independent experiments. (TIF) [file pone.0111777.s001.tif]

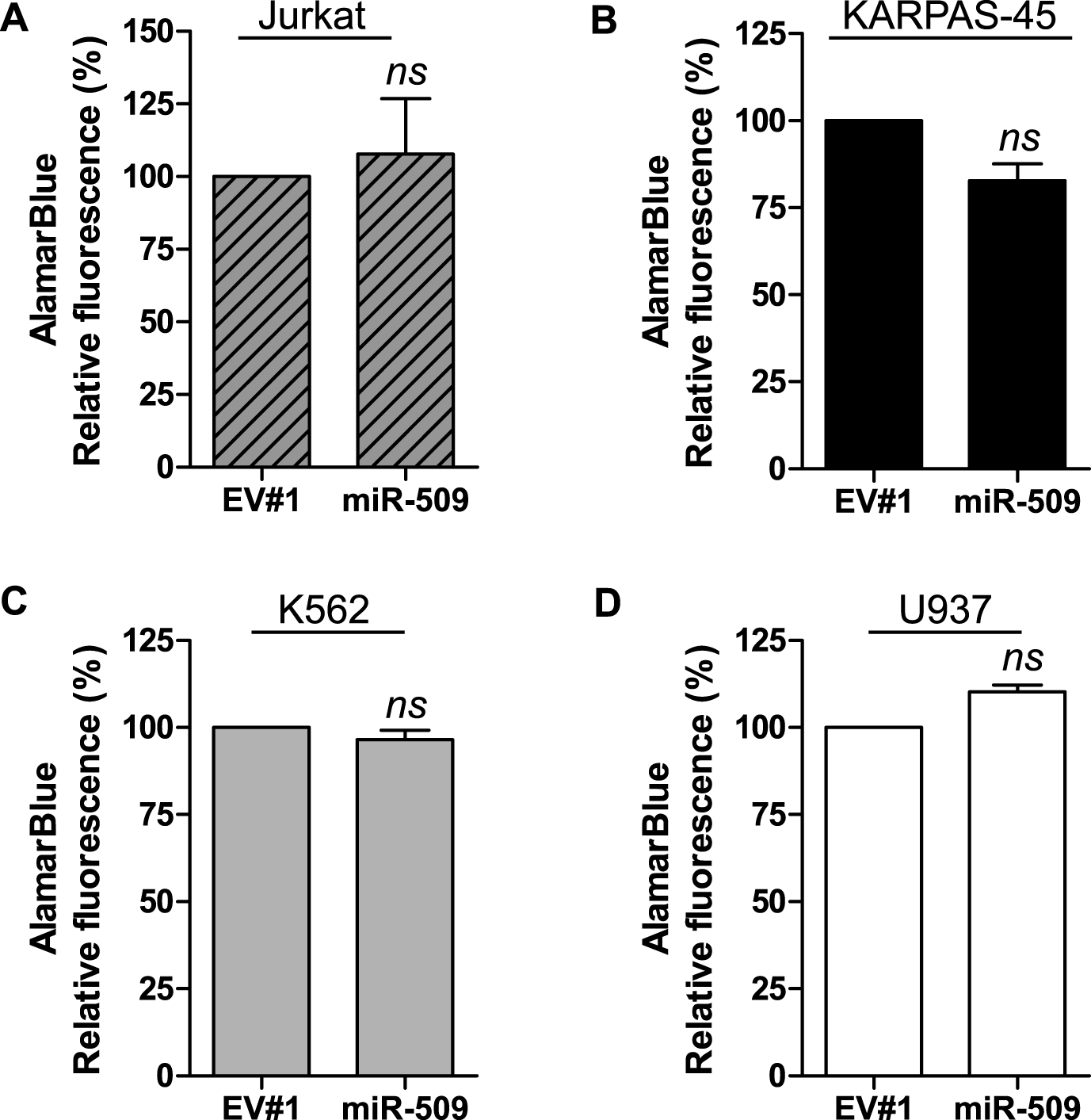

Supplement: Figure S2 — MiR-509 does not regulate the growth of Jurkat, KARPAS-45 and K562 cells. AlamarBlue cell growth assay was then performed on day 7 after transduction of (A) Jurkat, (B) KARPAS-45 and (C) K562 cells with either miR-509 lentivirus or EV#1. Each cell line was transduced with the indicated lentivirus to MOI = 2. On day 3 after transduction, cells were seeded at the indicated numbers per well/100 µl media: Jurkat (5×10^3^ cells), KARPAS-45 (3×10^3^ cells) and K562 (1.25×10^3^ cells) in triplicates in 96-well plates. Values for miR-509 were normalized to EV#1. Means ± SEMs, ns = no statistical significance was detected by Student's t test. (TIF) [file pone.0111777.s002.tif]

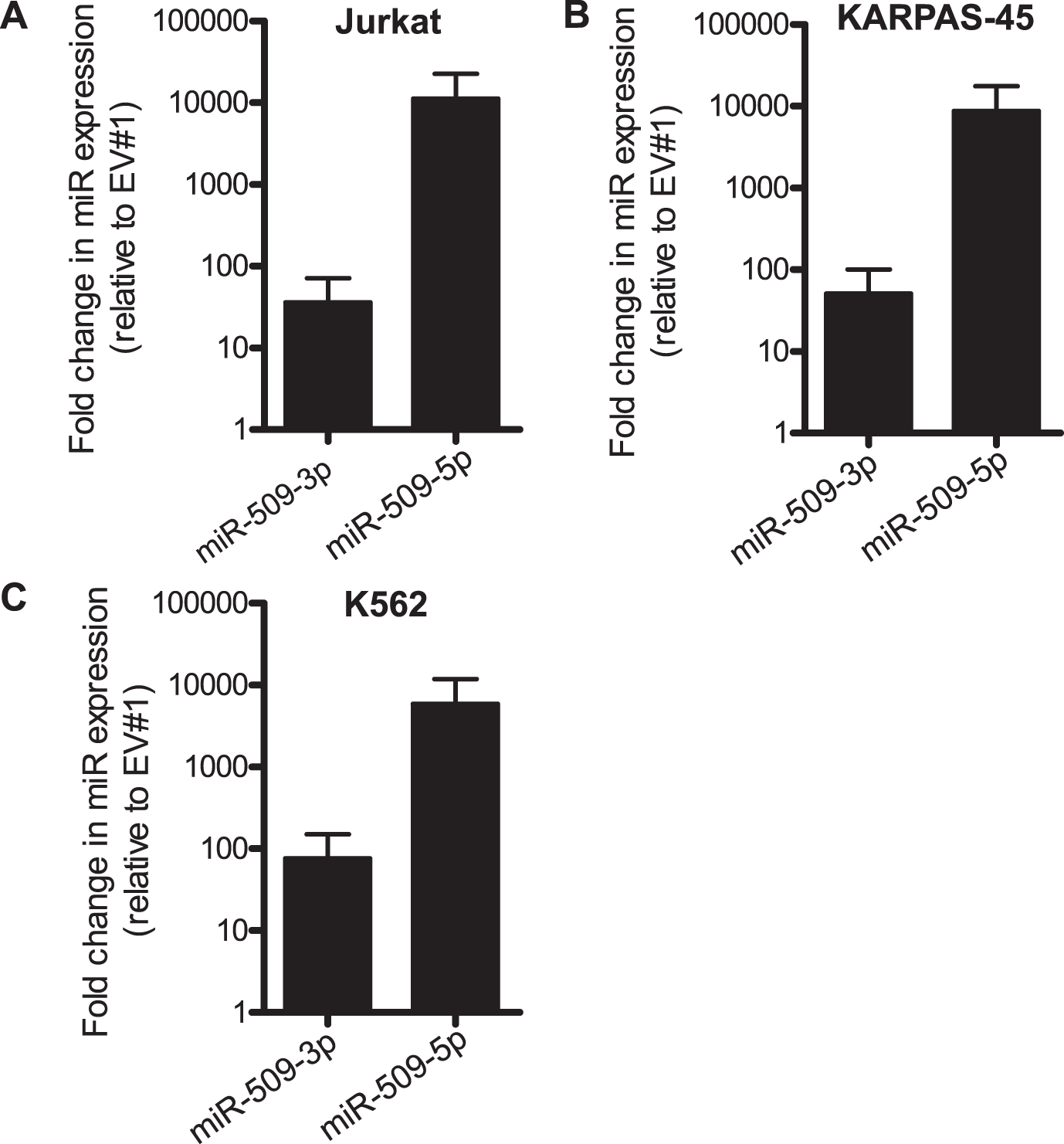

Supplement: Figure S3 — Enforced expression of miR-509 was detected by qRT-PCR in selected T-ALL and myeloid leukemia cell lines transduced with miR-509 lentivirus. (A) Jurkat, (B) KARPAS-45 and (C) K562 cells were transduced with miR-509 lentivirus or EV#1. On day 7 after transduction, cells were collected for RNA isolation. U18 was used as the endogenous control. Values shown were calculated as fold overexpression relative to each EV#1-transduced cells. Means ± SEMs are shown for 3 independent experiments. (TIF) [file pone.0111777.s003.tif]

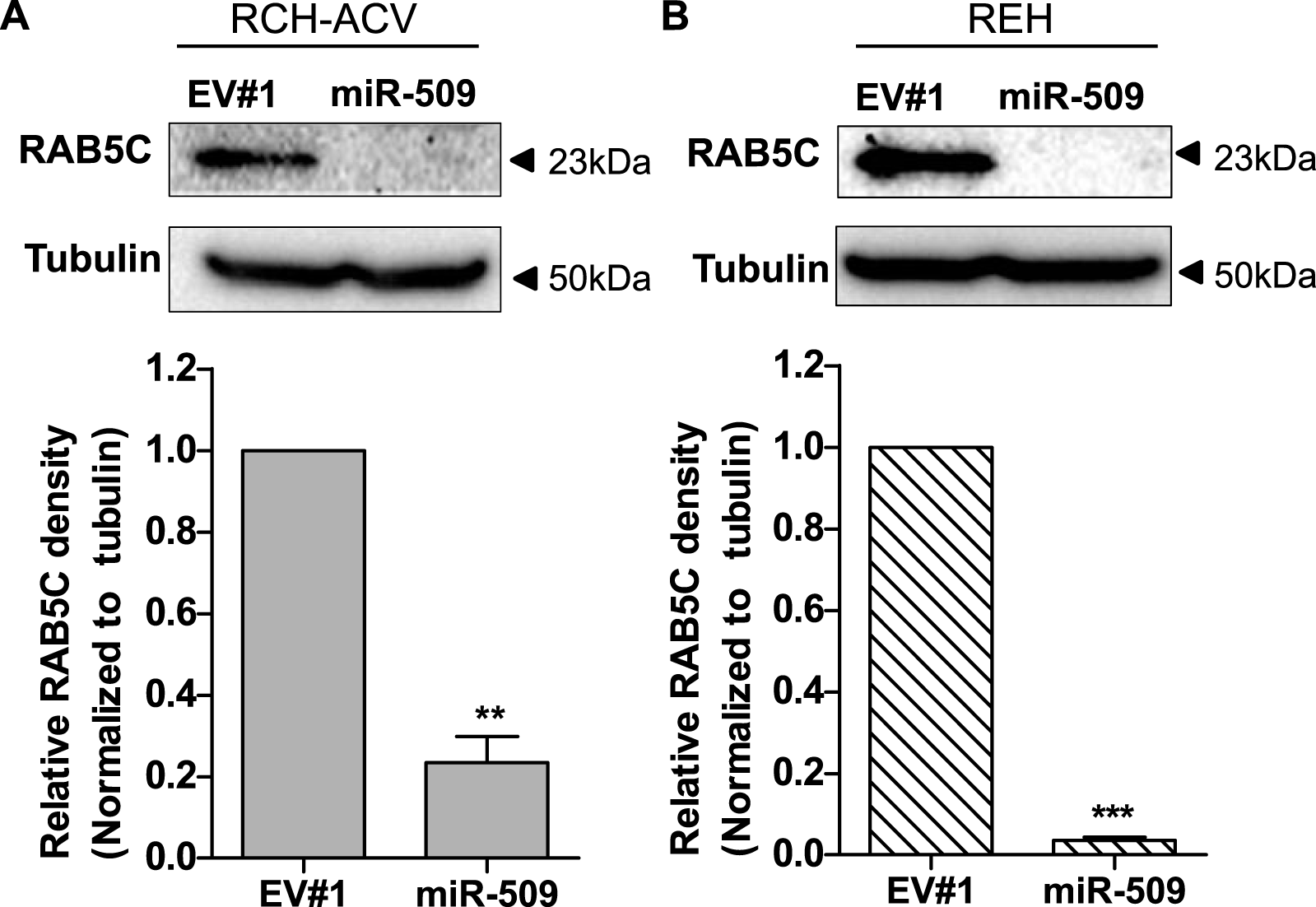

Supplement: Figure S4 — RAB5C protein levels were decreased in RCH-ACV and REH cells with enforced miR-509 expression. Representative western blot of RAB5C expression in (A) RCH-ACV and (B) REH. Cells were transduced with either EV#1or miR-509 overexpressing lentivirus, and whole cell lysates were harvested at 7 days after transduction. α-tubulin was used for loading control. The bar graph below represents the densitometry analysis of RAB5C expression of 3 independent experiments, normalized to α-tubulin, and relative densitometry was then calculated compared to EV#1. Data shown represent means ± SEMs, with statistical analysis by Student's t test. **p<0.01, ***p<0.001. (TIF) [file pone.0111777.s004.tif]

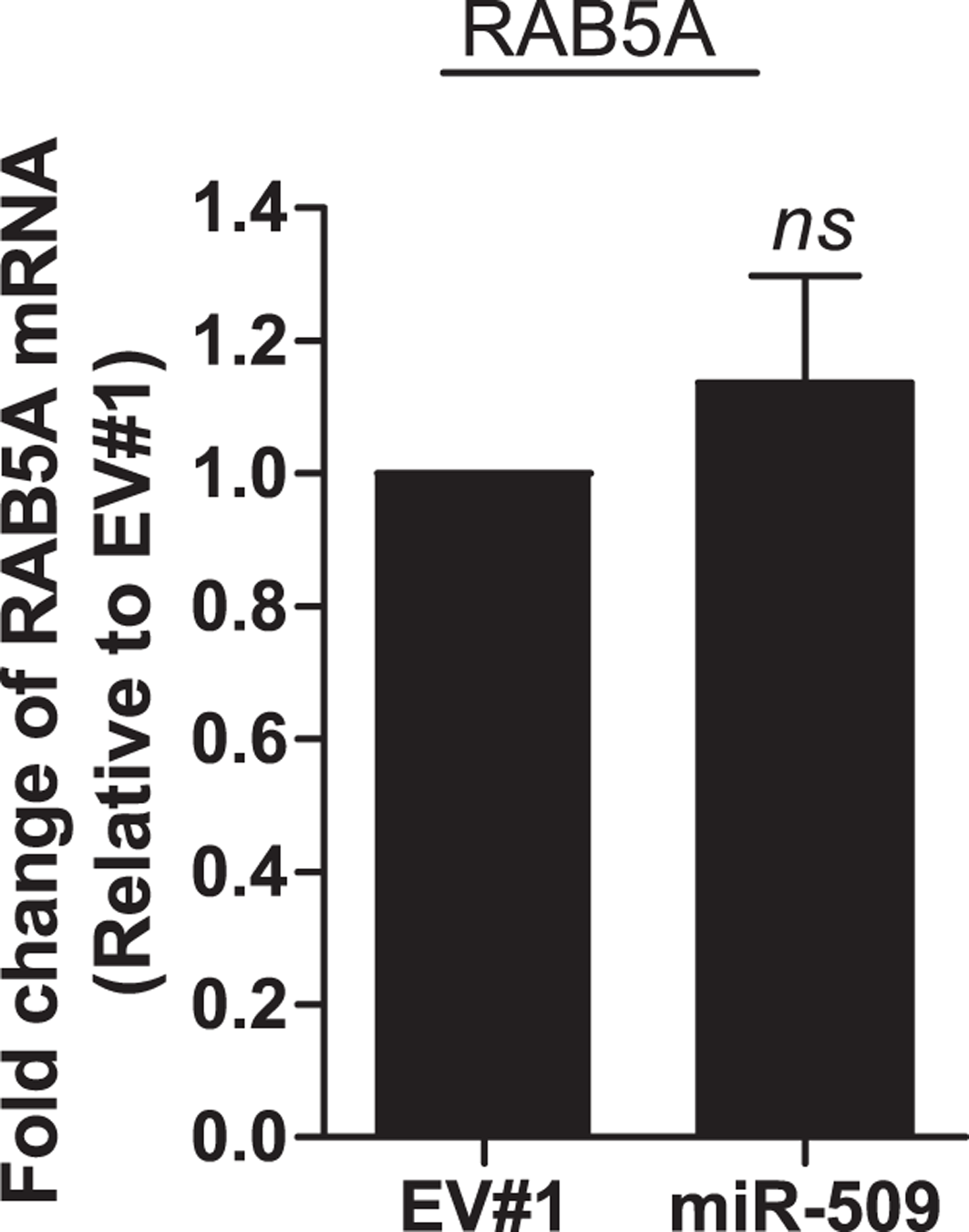

Supplement: Figure S5 — RAB5A mRNA levels show no change in miR-509-transduced NALM6 cells. NALM6 cells were transduced with empty vector #1 (EV#1) to MOI = 2, and RNA was isolated at day 7 after transduction for qRT-PCR. All values were normalized to GAPDH and fold-change was calculated relative to EV#1 sample. Data represents means ± SEMs of 3 independent experiments, with statistical analysis by Student's t test, ns = no statistical significance was detected by Student's t test. (TIF) [file pone.0111777.s005.tif]

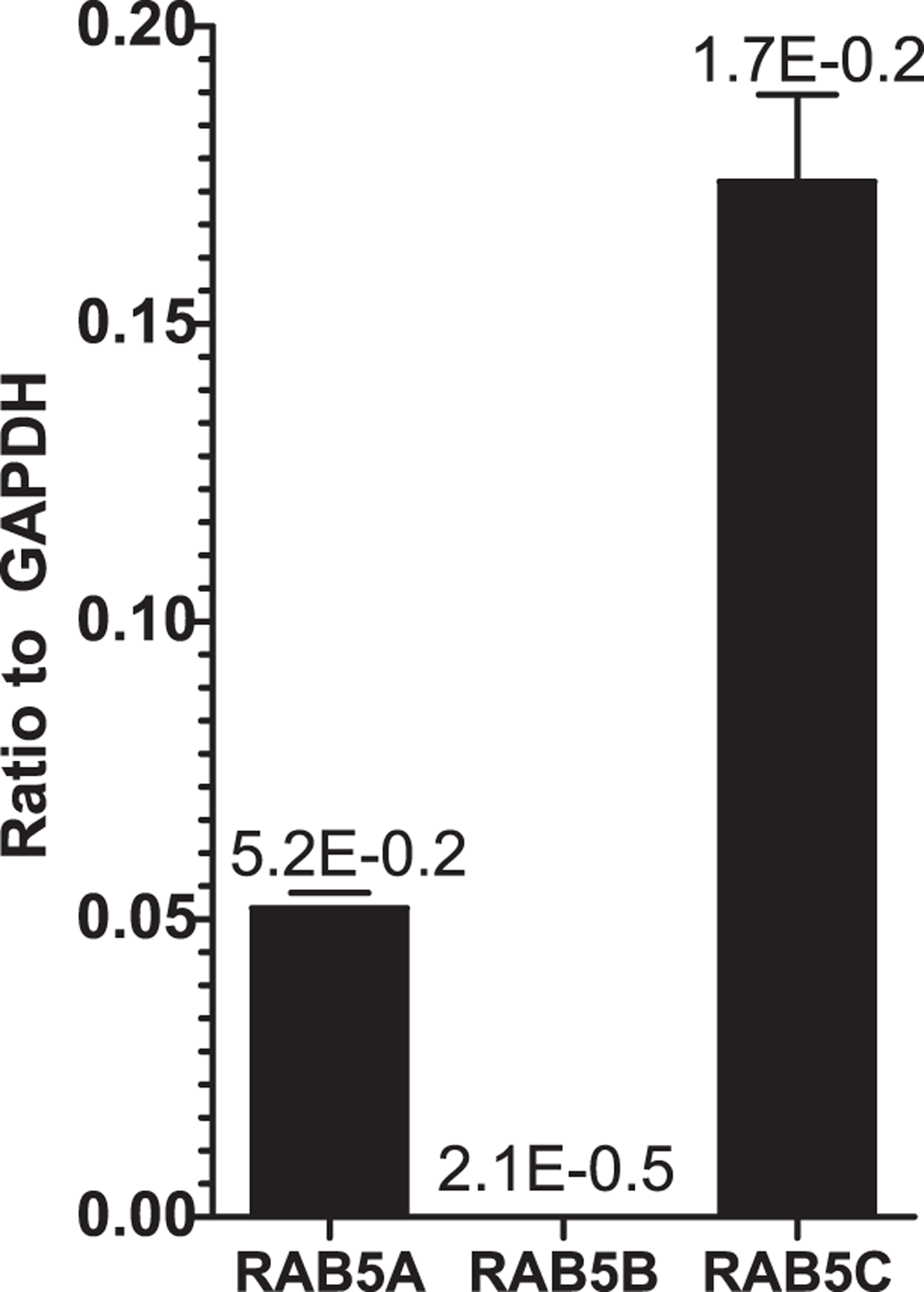

Supplement: Figure S6 — Expression of RAB5A and RAB5C, but not RAB5B, was detected in NALM6 cells using qRT-PCR. NALM6 cells were transduced with empty vector #1 (EV#1) to MOI = 2, and RNA was isolated at day 7 after transduction for qRT-PCR. Ratio to GAPDH (endogenous control) was calculated as 2E[-(RAB5Ct – GAPDHCt)]. Means ± SEMs, n = 3 independent experiments. Value above each bar represents the mean. (TIF) [file pone.0111777.s006.tif]

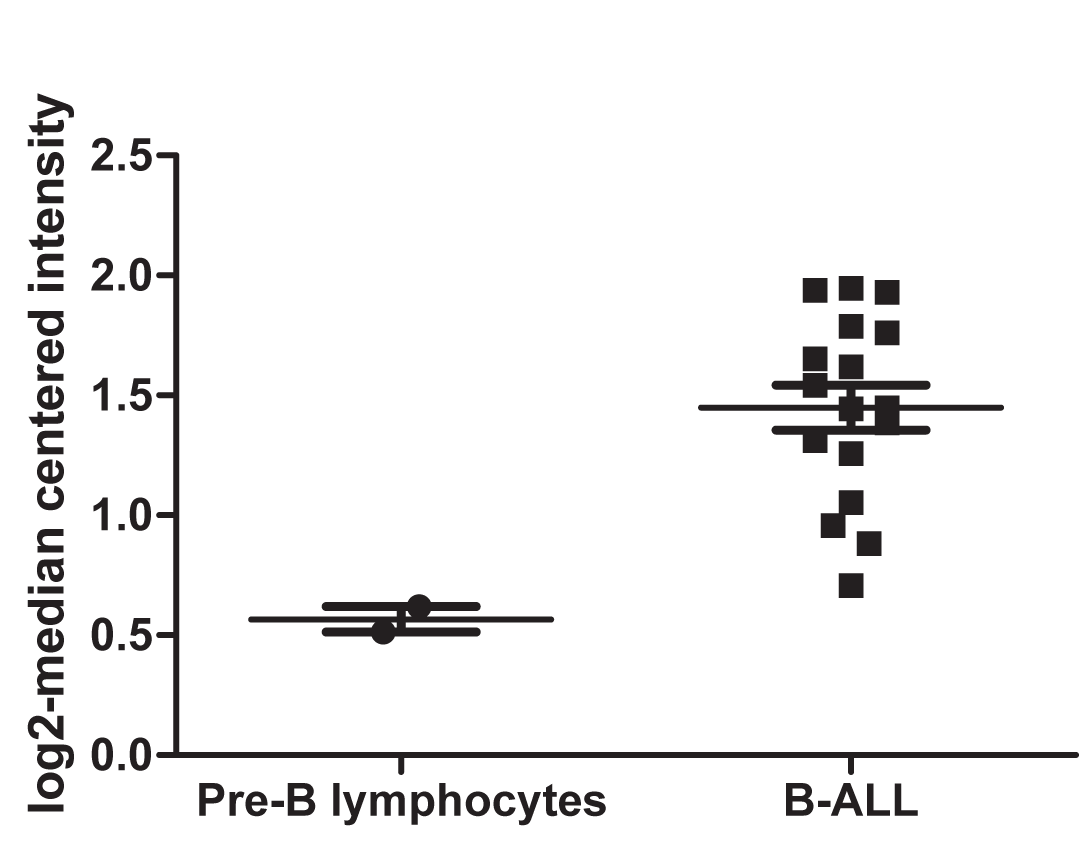

Supplement: Figure S7 — Scatter dot plot of RAB5C mRNA expression in B-ALL cells and pre-B lymphocytes based on Oncomine cancer microarray database. RAB5C expression in leukemia was examined using the Oncomine cancer microarray database by comparing specifically the ‘cancer versus normal’ analysis and setting a threshold of p-value ≤0.001 and 1.5-fold over-expression. 14 ‘cancer versus normal’ datasets were identified and we focused solely on leukemia in Oncomine. RAB5C was overexpressed by 1.8-fold (average; Student's t test, p = 3.67−6) in the dataset of B-ALL patient samples harboring the t(12;21) chromosomal translocation (producing the TEL/AML-1 fusion protein oncogene; n = 17) as compared to normal B-lymphoid precursors (pro/pre–B cells and immature B cells; n = 2) from healthy donors [67]. Error bars represent the mean ± SEM. (TIF) [file pone.0111777.s007.tif]
