# Supplementary material for: Regulation of RAB5C Is Important for the Growth Inhibitory Effects of MiR-509 in Human Precursor-B Acute Lymphoblastic Leukemia
Source: PLoS One. 2014 Nov 4;9(11):e111777. doi: 10.1371/journal.pone.0111777 (PMC4219775; doi:10.1371/journal.pone.0111777)
Supplement: Table S1 — List of primers used for cloning of miR hairpin with flanking genomic sequences. PCR products were first cloned into pJET1.2 and subcloned into empty lentiviral vector #1 (EV#1; pWCC52) downstream of GFP. MiR-509 was then subcloned from pWCC52-miR-509 into empty lentiviral vector #2 (EV#2; pWCC72) downstream of DsRed. (DOCX) [file pone.0111777.s008.docx]

**Supporting Table S1: List of primers used for cloning of miR hairpin with flanking genomic sequences.**

| **Human miR** | **Direction** | **Sequence (5' - 3')** |
| --- | --- | --- |
| miR-509-2 | Fwd | atcgatgatatcAGGACTCAAAGCGGGAAATA |
|  | Rev | acgcgtgtttaaacGGTCAGGCACCTGAGAGAGT |
| miR-432~136 | Fwd | CTCGAGGATATCtctgcggttgagtgctcg |
|  | Rev | GAATTCGGTACCgtccttgagaccctgatact |
| miR-873 | Fwd | ATCGATCTCGAGGATATCTGCCCACAAAACTGTTTGAA |
|  | Rev | ACGCGTGAATTCCGTACGCATCCATGTTGAGTGGACAAG |
| miR-381 | Fwd | ATCGATCTCGAGGATATCCTTTGGGATGGAGAGCTGAC |
|  | Rev | ACGCGTGAATTCGGTACCGCCTTCCCTAAAGTCATCCA |
| miR-550a-1 | Fwd | ATCGATCTCGAGCCCGGGCCTGTGTCATGGAAGTTT |
|  | Rev | ACGCGTGAATTCGGTACCGGGCAATTCTCCTTAAAACC |

PCR products were first cloned into pJET1.2 and subcloned into empty lentiviral vector #1 (EV#1; pWCC52) downstream of GFP. MiR-509 was then subcloned from pWCC52-miR-509 into empty lentiviral vector #2 (EV#2; pWCC72) downstream of DsRed.
