# Supplementary material for: Regulation of RAB5C Is Important for the Growth Inhibitory Effects of MiR-509 in Human Precursor-B Acute Lymphoblastic Leukemia
Source: PLoS One. 2014 Nov 4;9(11):e111777. doi: 10.1371/journal.pone.0111777 (PMC4219775; doi:10.1371/journal.pone.0111777)
Supplement: Table S2 — Primers used for PCR of RAB5C-3′UTR and deletion of miR-509-3p binding sites. Full length RAB5C-3′UTR was cloned into pmirGLO Dual-Luciferase miRNA Target Expression vector (Promega). This plasmid was then used as a template for site-directed mutagenesis to delete the first miR-509-3p binding sites in RAB5C-3′UTR-luciferase deletion construct, Δ1or Δ1Δ2 using primers Del56-72. For the deletion of the second miR-509-3p binding site in RAB5C-3′UTR-luciferase deletion construct, Δ2 or Δ1Δ2, standard PCR was performed using the Del758-767 primers. (DOCX) [file pone.0111777.s009.docx]

**Supporting Table S2: Primers used for PCR of RAB5C-3’UTR and deletion of miR-509-3p binding sites.**

| **Primers** | **Sequence (5’ to 3’)** |
| --- | --- |
| *Nhe*I-WT-RAB5C 3’UTR-Fwd | gccGCTAGCgccccccttgcctgcccg |
| WT-RAB5C 3’UTR-*Sbf*I-Rev | ggcCCTGCAGGgaggtcgatcgatcggct |
| Deletion of miR-509-3p binding site (Del56-72)-Sense | tgaatgacccgactggagcacttaacgactcg |
| Deletion of miR-509-3p binding site (Del56-72)-Antisense | cgagtcgttaagtgctccagtcgggtcattca |
| Deletion of miR-509-3p binding site (Del 758-767)-Fwd | gccGCTAGCgccccccttgcctgcccg |
| Deletion of miR-509-3p binding site (Del 758-767)-*Sbf*I-Rev | ggccctgcagggaggtcgatcgatcggctgactatattgacaagataacatgttgaagaaaacatacaa |

Full length RAB5C-3’UTR was cloned into pmirGLO Dual-Luciferase miRNA Target Expression vector (Promega). This plasmid was then used as a template for site-directed mutagenesis to delete the first miR-509-3p binding sites in RAB5C-3’UTR-luciferase deletion construct, Δ1or Δ1Δ2 using primers Del56-72. For the deletion of the second miR-509-3p binding site in RAB5C-3’UTR-luciferase deletion construct, Δ2 or Δ1Δ2, standard PCR was performed using the Del758-767 primers.
