# Supplementary material for: Regulation of RAB5C Is Important for the Growth Inhibitory Effects of MiR-509 in Human Precursor-B Acute Lymphoblastic Leukemia
Source: PLoS One. 2014 Nov 4;9(11):e111777. doi: 10.1371/journal.pone.0111777 (PMC4219775; doi:10.1371/journal.pone.0111777)
Supplement: Table S3 — Primers used in cloning of RAB5C lacking its 3′UTR into pWCC61 lentiviral vector (Empty lentiviral vector #3, EV#3). (DOCX) [file pone.0111777.s010.docx]

**Supporting Table S3: Primers used in cloning of RAB5C lacking its 3’UTR into pWCC61 lentiviral vector (Empty lentiviral vector #3, EV#3).**

| **Primers** | **Sequence (5’-3’)** |
| --- | --- |
| *Nhe*I-RAB5C-no 3’UTR-Fwd | gccgctagcATGGAACTGAGTTGGAGGTCCCCCTCC |
| RAB5C-no 3’UTR-*BamH*I-Rev | ggcggatccTCAGTTGCTGCAGCACTGGCTCC |
