# Supplementary material for: Regulation of RAB5C Is Important for the Growth Inhibitory Effects of MiR-509 in Human Precursor-B Acute Lymphoblastic Leukemia
Source: PLoS One. 2014 Nov 4;9(11):e111777. doi: 10.1371/journal.pone.0111777 (PMC4219775; doi:10.1371/journal.pone.0111777)
Supplement: Table S4 — List of primers used for SYBRGreen qRT-PCR. Primer sequences were obtained from PrimerBank. Fwd: Forward; Rev: Reverse. (DOCX) [file pone.0111777.s011.docx]

**Supporting Table S4: List of primers used for SYBRGreen qRT-PCR.**

| **Gene** | **Sequence (5'- 3')** |
| --- | --- |
| ERLIN2 Fwd | TCCACCACGAACTGAACCAG |
| ERLIN2 Rev | AACAGCTCAATGTAGACCTCTTG |
| FLI1 Fwd | CAGCCCCACAAGATCAACCC |
| FLI1 Rev | CACCGGAGACTCCCTGGAT |
| FOXP1 Fwd | GAAGGCCACAAAAGATCAGTG |
| FOXP1 Rev | TGGAAGCGGTAGTGTATAGAGG |
| GADPH Fwd | ACATCGCTCAGACACCATG |
| GADPH Rev | TGTAGTTGAGGTCAATGAAGGG |
| MAML1 Fwd | GACTCTCTCAACAAAAAGCGTCT |
| MAML1 Rev | AGGAAATGACTCACTGGGGTTA |
| PGRMC1 Fwd | GGGCTGCTGCATGAGATTTTC |
| PGRMC1 Rev | CCGCGCACGATCTTGTAGA |
| RAB5A Fwd | AGACCCAACGGGCCAAATAC |
| RAB5A Rev | GCCCCAATGGTACTCTCTTGAA |
| RAB5B Fwd | GAGCCCCAGGAGTGTTGAAG |
| RAB5B Rev | ACGTAATACCAGGCTTGACTTTC |
| RAB5C Fwd | CCGCTTTGTCAAGGGACAGTT |
| RAB5C Rev | AGGCTGTGATACCGCTCCT |
| RAC1 Fwd | ATGTCCGTGCAAAGTGGTATC |
| RAC1 Rev | CTCGGATCGCTTCGTCAAACA |
| TFDP2 Fwd | CTGCCTACCAATTCTGCTCAG |
| TFDP2 Rev | CGCTTCTGCTTTATCCGTTCT |
| UHMK1 Fwd | ACGCTGTCTGTTGCTTGAACT |
| UHMK1 Rev | GGCACAATGCTGTATCATCCAC |
| USP9X Fwd | TCGGAGGGAATGACAACCAG |
| USP9X Rev | GGAGTTGCCGGGGAATTTTCA |
| YWHAB Fwd | CATGAAGGCAGTCACAGAACA |
| YWHAB Rev | CTCACGGTACTCTTTGCCCAT |
| YWHAG Fwd | AGCCACTGTCGAATGAGGAAC |
| YWHAG Rev | CTGCTCAATGCTACTGATGACC |

Primer sequences were obtained from PrimerBank. Fwd: Forward; Rev: Reverse.
