# Supplementary material for: Regulation of RAB5C Is Important for the Growth Inhibitory Effects of MiR-509 in Human Precursor-B Acute Lymphoblastic Leukemia
Source: PLoS One. 2014 Nov 4;9(11):e111777. doi: 10.1371/journal.pone.0111777 (PMC4219775; doi:10.1371/journal.pone.0111777)
Supplement: Table S5 — List of TaqMan microRNA assay ID used for qRT-PCR. (DOCX) [file pone.0111777.s012.docx]

**Supporting Table S5: List of TaqMan microRNA assay ID used for qRT-PCR**

| **miR/U18** | **TaqMan MicroRNA assay ID (Life Technologies)** |
| --- | --- |
| miR-509-5p | 002235 |
| miR-509-3p | 002236 |
| miR-550a-3p | 001544 |
| miR-873-5p | 002356 |
| miR-381-3p | 000571 |
| miR-432-5p | 001026 |
| miR-136-5p | 000592 |
| U18 | 001204 |
