# Supplementary material for: Regulation of RAB5C Is Important for the Growth Inhibitory Effects of MiR-509 in Human Precursor-B Acute Lymphoblastic Leukemia
Source: PLoS One. 2014 Nov 4;9(11):e111777. doi: 10.1371/journal.pone.0111777 (PMC4219775; doi:10.1371/journal.pone.0111777)
Supplement: Table S6 — List of the 395 predicted targets of miR-509-5p and/or miR-509-3p selected based on filtering strategy shown in Figure 4A . These targets were subjected to a filtering strategy presented in Fig. 4A and meet the following criteria: (i) They are predicted targets of miR-509-5p and/or miR-509-3p from TargetScan6.2 and/or miRDB. (ii) These targets are not targets of miR-381, miR-550a, miR-873 and miR-432 as predicted by TargetScan6.2 and/or miRDB. (iii) These targets are expressed in NALM6 cells as determined by genome-wide microarray profiling downloaded from the Cancer Cell Line Encyclopedia and its expression levels are denoted in the microarray dataset as “marginal” or “present”. (DOCX) [file pone.0111777.s013.docx]

**Supporting Table S6. List of** **the 395 predicted targets of miR-509-5p and/or miR-509-3p selected based on filtering strategy shown in Figure 4A**.

| **Official symbol** | **Targeted by** | **Predicted by** |
| --- | --- | --- |
| AAK1 | miR-509-5p | TargetScan6.2 |
| ABCB10 | miR-509-3p | miRDB |
| ABL2 | miR-509-5p | miRDB |
| ACIN1 | miR-509-5p | TargetScan6.2 |
| ACTR1A | miR-509-5p | TargetScan6.2/miRDB |
| AK4 | miR-509-5p | miRDB |
| ALS2 | miR-509-5p | miRDB |
| AMOTL1 | miR-509-5p | miRDB |
| ANKH | miR-509-5p | TargetScan6.2/miRDB |
| ANKMY2 | miR-509-5p | miRDB |
| ANKRD10 | miR-509-5p | miRDB |
| ANKS1A | miR-509-3p | TargetScan6.2 |
| ANLN | miR-509-5p | TargetScan6.2 |
| AP3M1 | miR-509-5p | TargetScan6.2 |
| APBB2 | miR-509-5p | miRDB |
| ARF3 | miR-509-5p | TargetScan6.2 |
| ARF6 | miR-509-5p | miRDB |
| ARFGAP1 | miR-509-5p | miRDB |
| ARFGEF2 | miR-509-5p | TargetScan6.2 |
| ARHGAP1 | miR-509-3p | TargetScan6.2/miRDB |
| ARHGEF9 | miR-509-5p | TargetScan6.2 |
| ARID1A | miR-509-5p | TargetScan6.2 |
| ARID5B | miR-509-3p | TargetScan6.2 |
| ASB14 | miR-509-5p | miRDB |
| ASCC3 | miR-509-5p | miRDB |
| ASGR1 | miR-509-5p | miRDB |
| ATAD2B | miR-509-5p | TargetScan6.2 |
| ATP11A | miR-509-3p | miRDB |
| ATP11C | miR-509-5p | miRDB |
| ATP5L | miR-509-3p | TargetScan6.2 |
| ATP8A1 | miR-509-5p | miRDB |
| ATXN1L | miR-509-5p | TargetScan6.2 |
| ATXN7 | miR-509-5p | TargetScan6.2 |
| BACH2 | miR-509-5p | TargetScan6.2 |
| BBS12 | miR-509-5p | miRDB |
| BDH2 | miR-509-3p | miRDB |
| BMPR2 | miR-509-5p/3p | TargetScan6.2 |
| BRD2 | miR-509-5p | miRDB |
| BRP44L | miR-509-5p | miRDB |
| BRWD3 | miR-509-3p | TargetScan6.2 |
| BTRC | miR-509-5p | TargetScan6.2 |
| BUB3 | miR-509-5p | miRDB |
| C11orf95 | miR-509-5p | miRDB |
| C14orf109 | miR-509-5p | miRDB |
| C14orf45 | miR-509-3p | miRDB |
| C15orf17 | miR-509-5p | TargetScan6.2 |
| C15orf29 | miR-509-3p | TargetScan6.2/miRDB |
| C17orf75 | miR-509-5p | TargetScan6.2 |
| C20orf11 | miR-509-5p | TargetScan6.2/miRDB |
| C20orf160 | miR-509-5p | TargetScan6.2 |
| C20orf194 | miR-509-5p | TargetScan6.2 |
| C20orf197 | miR-509-3p | miRDB |
| C2orf43 | miR-509-5p | miRDB |
| C4orf46 | miR-509-3p | miRDB |
| CACNB4 | miR-509-3p | miRDB |
| CALD1 | miR-509-5p | TargetScan6.2 |
| CALU | miR-509-3p | TargetScan6.2 |
| CAMTA1 | miR-509-5p | TargetScan6.2 |
| CAPN7 | miR-509-5p | miRDB |
| CASC3 | miR-509-5p | miRDB |
| CCDC99 | miR-509-5p | miRDB |
| CCNJ | miR-509-5p | miRDB |
| CCNYL1 | miR-509-5p | TargetScan6.2 |
| CDC14A | miR-509-5p | miRDB |
| CDK17 | miR-509-5p | miRDB |
| CDS2 | miR-509-5p | TargetScan6.2 |
| CENPBD1 | miR-509-5p | TargetScan6.2 |
| CEP152 | miR-509-5p | miRDB |
| CHM | miR-509-5p | TargetScan6.2 |
| CHMP7 | miR-509-5p | TargetScan6.2/miRDB |
| CHORDC1 | miR-509-5p | miRDB |
| CHST11 | miR-509-5p | TargetScan6.2 |
| CHSY1 | miR-509-5p | TargetScan6.2 |
| CIT | miR-509-5p | TargetScan6.2 |
| COPS2 | miR-509-5p | TargetScan6.2 |
| COQ7 | miR-509-5p | miRDB |
| CORO2B | miR-509-5p | miRDB |
| CREBZF | miR-509-5p | TargetScan6.2 |
| CRIPT | miR-509-5p | miRDB |
| CTNNA1 | miR-509-5p | miRDB |
| CTPS | miR-509-5p | miRDB |
| CTR9 | miR-509-5p | miRDB |
| DBT | miR-509-5p | miRDB |
| DCLRE1C | miR-509-5p | miRDB |
| DCP1A | miR-509-3p | TargetScan6.2 |
| DDX18 | miR-509-5p | miRDB |
| DDX23 | miR-509-5p | miRDB |
| DEDD | miR-509-3p | TargetScan6.2 |
| DHDDS | miR-509-3p | TargetScan6.2 |
| DLG3 | miR-509-3p | TargetScan6.2 |
| DNAJA1 | miR-509-5p | TargetScan6.2 |
| DNAJC11 | miR-509-5p | miRDB |
| DNAJC25 | miR-509-5p | miRDB |
| DNAJC3 | miR-509-5p | TargetScan6.2 |
| DNAJC5 | miR-509-5p | TargetScan6.2 |
| DOCK4 | miR-509-5p | TargetScan6.2 |
| DPYD | miR-509-5p | miRDB |
| DPYSL2 | miR-509-5p | TargetScan6.2/miRDB |
| DYNC2LI1 | miR-509-3p | TargetScan6.2 |
| E2F2 | miR-509-5p | miRDB |
| EEF2K | miR-509-5p | TargetScan6.2 |
| EHD4 | miR-509-5p | miRDB |
| EHMT1 | miR-509-5p | TargetScan6.2 |
| EIF1B | miR-509-5p | TargetScan6.2/miRDB |
| EIF4E3 | miR-509-5p | TargetScan6.2 |
| EIF5B | miR-509-5p | TargetScan6.2/miRDB |
| ELL | miR-509-5p | TargetScan6.2 |
| ENAH | miR-509-5p | TargetScan6.2/miRDB |
| ENPEP | miR-509-5p | miRDB |
| ENPP2 | miR-509-3p | miRDB |
| EPB41L5 | miR-509-5p | TargetScan6.2 |
| ERLIN2 | miR-509-5p/3p | miRDB |
| EXOC5 | miR-509-5p | miRDB |
| EXTL1 | miR-509-3p | TargetScan6.2 |
| FAM116A | miR-509-5p/3p | TargetScan6.2 |
| FAM120A | miR-509-5p | miRDB |
| FAM134B | miR-509-5p | miRDB |
| FAM13A | miR-509-5p | miRDB |
| FAM40A | miR-509-5p | TargetScan6.2 |
| FBXL5 | miR-509-3p | miRDB |
| FBXW11 | miR-509-5p | TargetScan6.2/miRDB |
| FHL1 | miR-509-5p | TargetScan6.2 |
| FKTN | miR-509-5p | miRDB |
| FLI1 | miR-509-3p | TargetScan6.2 |
| FNBP1 | miR-509-5p | TargetScan6.2 |
| FOXM1 | miR-509-5p | TargetScan6.2/miRDB |
| FOXP1 | miR-509-5p | TargetScan6.2/miRDB |
| G3BP2 | miR-509-5p | TargetScan6.2/miRDB |
| GAS7 | miR-509-3p | TargetScan6.2/miRDB |
| GATAD1 | miR-509-5p | miRDB |
| GEMIN5 | miR-509-5p | TargetScan6.2 |
| GFOD1 | miR-509-5p | miRDB |
| GFOD2 | miR-509-5p | miRDB |
| GMFB | miR-509-5p | TargetScan6.2/miRDB |
| GOLPH3 | miR-509-3p | miRDB |
| GOSR1 | miR-509-3p | TargetScan6.2/miRDB |
| GPI | miR-509-5p | miRDB |
| GPM6B | miR-509-5p | TargetScan6.2/miRDB |
| GPR155 | miR-509-5p | TargetScan6.2 |
| GPR63 | miR-509-5p | miRDB |
| GRB10 | miR-509-5p | miRDB |
| GTF2H5 | miR-509-5p | miRDB |
| HABP4 | miR-509-3p | miRDB |
| HERC4 | miR-509-5p | miRDB |
| HLA-DPA1 | miR-509-5p | miRDB |
| HMBS | miR-509-3p | miRDB |
| HNRNPU | miR-509-5p | miRDB |
| HNRNPUL1 | miR-509-5p | TargetScan6.2 |
| HOOK3 | miR-509-5p | miRDB |
| HSDL1 | miR-509-5p | miRDB |
| IBTK | miR-509-5p | miRDB |
| IDE | miR-509-5p | TargetScan6.2/miRDB |
| IDH1 | miR-509-5p | miRDB |
| IFT57 | miR-509-5p | miRDB |
| IL13RA1 | miR-509-3p | miRDB |
| IMPA1 | miR-509-5p | miRDB |
| IMPDH1 | miR-509-5p | TargetScan6.2 |
| INTS10 | miR-509-3p | miRDB |
| IPO8 | miR-509-5p | TargetScan6.2/miRDB |
| JARID2 | miR-509-5p | TargetScan6.2/miRDB |
| KDM4A | miR-509-5p | TargetScan6.2/miRDB |
| KDM4C | miR-509-5p | miRDB |
| KIAA0100 | miR-509-5p | TargetScan6.2 |
| KIAA0415 | miR-509-5p | miRDB |
| KIAA0754 | miR-509-3p | miRDB |
| KIAA1328 | miR-509-3p | miRDB |
| KIF15 | miR-509-5p | miRDB |
| KLHL18 | miR-509-5p | TargetScan6.2 |
| KPNA4 | miR-509-5p | TargetScan6.2/miRDB |
| LAMTOR3 | miR-509-3p | TargetScan6.2 |
| LETMD1 | miR-509-5p | TargetScan6.2 |
| LMLN | miR-509-5p | miRDB |
| LONP2 | miR-509-3p | miRDB |
| LRP8 | miR-509-5p | miRDB |
| LSM5 | miR-509-5p | miRDB |
| LYSMD4 | miR-509-5p | miRDB |
| MACF1 | miR-509-5p | TargetScan6.2 |
| MAML1 | miR-509-5p | TargetScan6.2/miRDB |
| MAML2 | miR-509-5p | miRDB |
| MAML3 | miR-509-5p | miRDB |
| MAP3K8 | miR-509-3p | miRDB |
| MAP4K3 | miR-509-5p | miRDB |
| MAPK1IP1L | miR-509-3p | miRDB |
| MARCH5 | miR-509-5p | miRDB |
| MBNL3 | miR-509-5p | TargetScan6.2 |
| MBOAT2 | miR-509-5p | miRDB |
| MCFD2 | miR-509-3p | miRDB |
| METTL6 | miR-509-5p | miRDB |
| METTL8 | miR-509-5p | TargetScan6.2 |
| METTL9 | miR-509-3p | miRDB |
| MFAP3 | miR-509-3p | TargetScan6.2 |
| MIER3 | miR-509-5p | TargetScan6.2 |
| MKLN1 | miR-509-5p | TargetScan6.2/miRDB |
| MMD | miR-509-5p | miRDB |
| MRPL19 | miR-509-5p | miRDB |
| MSL3 | miR-509-5p | miRDB |
| MTCP1 | miR-509-5p | TargetScan6.2 |
| MTHFD2 | miR-509-5p | TargetScan6.2 |
| MTMR3 | miR-509-3p | TargetScan6.2 |
| MYBL1 | miR-509-5p | miRDB |
| MYO5A | miR-509-5p | miRDB |
| N4BP1 | miR-509-5p | miRDB |
| NAA30 | miR-509-3p | miRDB |
| NDUFA9 | miR-509-5p | miRDB |
| NEO1 | miR-509-3p | TargetScan6.2 |
| NF1 | miR-509-5p | TargetScan6.2 |
| NFATC2IP | miR-509-5p | miRDB |
| NFXL1 | miR-509-5p | miRDB |
| NNT | miR-509-5p | miRDB |
| NR1D2 | miR-509-3p | TargetScan6.2 |
| NRIP1 | miR-509-5p | TargetScan6.2/miRDB |
| NSUN4 | miR-509-5p | miRDB |
| NUDT7 | miR-509-5p | miRDB |
| OGG1 | miR-509-5p | miRDB |
| ORMDL1 | miR-509-5p | miRDB |
| OSBP | miR-509-3p | TargetScan6.2/miRDB |
| OSCAR | miR-509-3p | miRDB |
| PAK2 | miR-509-5p | miRDB |
| PANK3 | miR-509-5p | TargetScan6.2 |
| PAPD5 | miR-509-3p | TargetScan6.2 |
| PAPD7 | miR-509-5p | TargetScan6.2 |
| PAQR3 | miR-509-5p | miRDB |
| PBLD | miR-509-3p | TargetScan6.2/miRDB |
| PBX3 | miR-509-3p | TargetScan6.2/miRDB |
| PCDHA9 | miR-509-3p | TargetScan6.2 |
| PDCD6IP | miR-509-5p | TargetScan6.2/miRDB |
| PELO | miR-509-3p | miRDB |
| PGRMC1 | miR-509-5p | TargetScan6.2/miRDB |
| PHACTR3 | miR-509-5p | miRDB |
| PHF13 | miR-509-5p | TargetScan6.2 |
| PHF20L1 | miR-509-3p | miRDB |
| PHF21A | miR-509-5p | TargetScan6.2 |
| PHLPP2 | miR-509-3p | TargetScan6.2/miRDB |
| PICALM | miR-509-5p | TargetScan6.2 |
| PIP4K2B | miR-509-5p | miRDB |
| PLDN | miR-509-5p | miRDB |
| PLEKHM1 | miR-509-5p | TargetScan6.2 |
| PODXL | miR-509-5p | miRDB |
| POGLUT1 | miR-509-3p | TargetScan6.2 |
| POLR1D | miR-509-5p | miRDB |
| POLR3F | miR-509-5p | miRDB |
| PPIA | miR-509-5p | TargetScan6.2/miRDB |
| PPM1F | miR-509-5p | TargetScan6.2 |
| PPP1R3B | miR-509-5p | miRDB |
| PQLC3 | miR-509-5p | miRDB |
| PRKCI | miR-509-5p | miRDB |
| PRR14L | miR-509-5p | TargetScan6.2 |
| PRRC2B | miR-509-5p | TargetScan6.2 |
| PRUNE | miR-509-3p | TargetScan6.2/miRDB |
| PSTPIP2 | miR-509-5p | TargetScan6.2/miRDB |
| PTEN | miR-509-3p | TargetScan6.2 |
| PTPN11 | miR-509-5p | TargetScan6.2/miRDB |
| PTPN22 | miR-509-3p | miRDB |
| PURB | miR-509-5p/3p | TargetScan6.2 |
| RAB11A | miR-509-5p | TargetScan6.2/miRDB |
| RAB11FIP5 | miR-509-5p | TargetScan6.2 |
| RAB14 | miR-509-5p | TargetScan6.2 |
| RAB23 | miR-509-5p | miRDB |
| RAB5B | miR-509-5p | TargetScan6.2 |
| RAB5C | miR-509-3p | TargetScan6.2/miRDB |
| RAB6A | miR-509-5p | TargetScan6.2/miRDB |
| RABL3 | miR-509-5p | TargetScan6.2 |
| RAC1 | miR-509-3p | TargetScan6.2/miRDB |
| RAD23B | miR-509-5p | TargetScan6.2 |
| RALGAPB | miR-509-5p | TargetScan6.2 |
| RANBP2 | miR-509-5p | TargetScan6.2 |
| RANBP6 | miR-509-5p | miRDB |
| RAP2C | miR-509-5p | TargetScan6.2/miRDB |
| RBM22 | miR-509-5p | miRDB |
| RGS2 | miR-509-5p | miRDB |
| RHOBTB3 | miR-509-3p | miRDB |
| RHOT1 | miR-509-5p | TargetScan6.2/miRDB |
| RIC8B | miR-509-5p | TargetScan6.2 |
| RIMKLA | miR-509-5p | miRDB |
| RIPK2 | miR-509-5p | miRDB |
| RIT1 | miR-509-5p | TargetScan6.2/miRDB |
| RNF130 | miR-509-3p | TargetScan6.2/miRDB |
| RNF139 | miR-509-5p | miRDB |
| RNF4 | miR-509-5p | miRDB |
| RNFT1 | miR-509-5p | miRDB |
| RNGTT | miR-509-5p | miRDB |
| RNMT | miR-509-5p | TargetScan6.2 |
| RPGRIP1L | miR-509-5p | TargetScan6.2/miRDB |
| RREB1 | miR-509-5p | TargetScan6.2 |
| RSBN1 | miR-509-5p | TargetScan6.2 |
| RUNX1 | miR-509-5p | TargetScan6.2 |
| RXRA | miR-509-3p | TargetScan6.2 |
| RXRB | miR-509-5p | miRDB |
| RYBP | miR-509-3p | miRDB |
| SACS | miR-509-5p | miRDB |
| SAMD13 | miR-509-5p | miRDB |
| SENP7 | miR-509-5p | TargetScan6.2 |
| SEPSECS | miR-509-5p | TargetScan6.2/miRDB |
| SEPT6 | miR-509-5p | miRDB |
| SETD4 | miR-509-5p | miRDB |
| SETD5 | miR-509-5p | TargetScan6.2 |
| SF3B4 | miR-509-3p | TargetScan6.2 |
| SFPQ | miR-509-3p | miRDB |
| SGCB | miR-509-3p | TargetScan6.2 |
| SGTB | miR-509-5p | TargetScan6.2 |
| SHISA5 | miR-509-5p | TargetScan6.2 |
| SLC12A6 | miR-509-3p | TargetScan6.2 |
| SLC15A2 | miR-509-5p | TargetScan6.2 |
| SLC25A38 | miR-509-5p | miRDB |
| SLC25A5 | miR-509-5p | miRDB |
| SLC31A1 | miR-509-5p | miRDB |
| SLC35A2 | miR-509-3p | miRDB |
| SLC38A9 | miR-509-5p | miRDB |
| SLC39A9 | miR-509-5p | miRDB |
| SLC7A6 | miR-509-5p | TargetScan6.2 |
| SMAD2 | miR-509-5p/3p | TargetScan6.2 |
| SNX13 | miR-509-3p | TargetScan6.2/miRDB |
| SOCS7 | miR-509-5p | TargetScan6.2 |
| SOS1 | miR-509-5p | miRDB |
| SPHK2 | miR-509-3p | TargetScan6.2 |
| SPRED2 | miR-509-5p | TargetScan6.2 |
| SRSF11 | miR-509-5p | TargetScan6.2/miRDB |
| SRSF4 | miR-509-5p | TargetScan6.2 |
| SSH1 | miR-509-5p | TargetScan6.2 |
| ST13 | miR-509-5p | TargetScan6.2 |
| ST3GAL2 | miR-509-3p | TargetScan6.2/miRDB |
| ST3GAL3 | miR-509-3p | TargetScan6.2 |
| STAT5A | miR-509-5p | miRDB |
| STS | miR-509-5p | miRDB |
| SUPT16H | miR-509-3p | TargetScan6.2 |
| SYNJ2BP | miR-509-5p | miRDB |
| SYS1 | miR-509-3p | miRDB |
| TAB2 | miR-509-5p | TargetScan6.2/miRDB |
| TAF9B | miR-509-5p | miRDB |
| TARDBP | miR-509-5p | TargetScan6.2 |
| TBC1D1 | miR-509-5p | miRDB |
| TBCC | miR-509-5p | miRDB |
| TCF12 | miR-509-5p | TargetScan6.2 |
| TCF7L2 | miR-509-3p | TargetScan6.2/miRDB |
| TET1 | miR-509-5p/3p | TargetScan6.2 |
| TFDP2 | miR-509-5p | TargetScan6.2/miRDB |
| THUMPD3 | miR-509-5p | miRDB |
| TM9SF3 | miR-509-5p | TargetScan6.2 |
| TMEM109 | miR-509-5p | TargetScan6.2 |
| TMEM143 | miR-509-3p | TargetScan6.2 |
| TMEM178 | miR-509-5p | TargetScan6.2 |
| TMEM203 | miR-509-5p | miRDB |
| TMEM209 | miR-509-5p | miRDB |
| TMOD3 | miR-509-3p | miRDB |
| TMX4 | miR-509-5p | TargetScan6.2 |
| TNPO1 | miR-509-5p | TargetScan6.2 |
| TOM1L1 | miR-509-5p | miRDB |
| TOMM70A | miR-509-5p | miRDB |
| TP53INP1 | miR-509-3p | TargetScan6.2/miRDB |
| TPMT | miR-509-3p | miRDB |
| TRADD | miR-509-5p | miRDB |
| TRIM2 | miR-509-5p | miRDB |
| TRIM39 | miR-509-5p | TargetScan6.2/miRDB |
| TRIM52 | miR-509-5p | miRDB |
| TRIM59 | miR-509-5p | miRDB |
| TSC1 | miR-509-5p | TargetScan6.2 |
| TTC17 | miR-509-3p | TargetScan6.2/miRDB |
| TTC28 | miR-509-5p | TargetScan6.2 |
| TTF2 | miR-509-5p | miRDB |
| TTL | miR-509-5p | miRDB |
| TYW3 | miR-509-5p | miRDB |
| UGP2 | miR-509-5p | miRDB |
| UHMK1 | miR-509-5p | TargetScan6.2/miRDB |
| UNC13B | miR-509-3p | miRDB |
| UQCR11 | miR-509-5p | miRDB |
| USP27X | miR-509-5p/3p | TargetScan6.2 |
| USP37 | miR-509-5p | miRDB |
| USP38 | miR-509-5p | TargetScan6.2 |
| USP47 | miR-509-3p | TargetScan6.2/miRDB |
| USP9X | miR-509-5p | TargetScan6.2/miRDB |
| VAMP7 | miR-509-5p | miRDB |
| VCP | miR-509-5p | miRDB |
| VGLL4 | miR-509-5p | TargetScan6.2 |
| VIM | miR-509-5p | TargetScan6.2 |
| VPS36 | miR-509-3p | TargetScan6.2 |
| WBP5 | miR-509-3p | miRDB |
| WBSCR22 | miR-509-5p | miRDB |
| WDFY1 | miR-509-5p | TargetScan6.2/miRDB |
| WDR20 | miR-509-5p | TargetScan6.2 |
| WDR5B | miR-509-3p | miRDB |
| WDR82 | miR-509-5p | TargetScan6.2 |
| WDTC1 | miR-509-5p | TargetScan6.2 |
| YWHAB | miR-509-5p | TargetScan6.2 |
| YWHAG | miR-509-5p | TargetScan6.2/miRDB |
| ZBTB11 | miR-509-5p | miRDB |
| ZCCHC17 | miR-509-5p | miRDB |
| ZFP106 | miR-509-5p | miRDB |
| ZHX1 | miR-509-5p | miRDB |
| ZNF107 | miR-509-3p | miRDB |
| ZNF345 | miR-509-3p | miRDB |
| ZNF423 | miR-509-3p | TargetScan6.2/miRDB |
| ZNF551 | miR-509-5p | miRDB |
| ZNF641 | miR-509-5p | miRDB |
| ZNF777 | miR-509-3p | TargetScan6.2 |
| ZNF84 | miR-509-5p | miRDB |

These targets were subjected to a filtering strategy presented in Fig. 4A and meet the following criteria: (i) They are predicted targets of miR-509-5p and/or miR-509-3p from TargetScan6.2 and/or miRDB. (ii) These targets are not targets of miR-381, miR-550a, miR-873 and miR-432 as predicted by TargetScan6.2 and/or miRDB. (iii) These targets are expressed in NALM6 cells as determined by genome-wide microarray profiling downloaded from the Cancer Cell Line Encyclopedia and its expression levels are denoted in the microarray dataset as “marginal” or “present”.
